# In Search of Synergistic Insect Repellents: Modeling of Muscarinic GPCR Interactions with Classical and Bitopic Photoactive Ligands

**DOI:** 10.3390/molecules27103280

**Published:** 2022-05-20

**Authors:** Beata Niklas, Bruno Lapied, Wieslaw Nowak

**Affiliations:** 1 Institute of Physics, Faculty of Physics, Astronomy and Informatics, Nicolaus Copernicus University in Torun, 87-100 Torun, Poland; beata.niklas@doktorant.umk.pl; 2 Faculty of Biological and Veterinary Sciences, Nicolaus Copernicus University, Lwowska 1, 87-100 Torun, Poland; 3 University Angers, INRAE, SIFCIR, SFR QUASAV, F-49045 Angers, France; bruno.lapied@univ-angers.fr

**Keywords:** GPCR modulators, docking, bitopic ligands, molecular dynamics, IR3535, DEET, muscarinic receptors, synergy

## Abstract

Insect vector-borne diseases pose serious health problems, so there is a high demand for efficient molecules that could reduce transmission. Using molecular docking and molecular dynamics (MD) simulation, we studied a series of compounds acting on human and insect muscarinic acetylcholine receptors (mAChRs), a novel target of synergistic agents in pest control. We characterized early conformational changes of human M1 and fruit fly type-A mAChR G protein-coupled receptors (GPCRs) in response to DEET, IR3535, and muscarine binding based on the MD analysis of the activation microswitches known to form the signal transduction pathway in class A GPCRs. We indicated groups of microswitches that are the most affected by the presence of a ligand. Moreover, to increase selectivity towards insects, we proposed a new, bitopic, photoswitchable mAChR ligand—BQCA-azo-IR353 and studied its interactions with both receptors. Modeling data showed that using a bitopic ligand may be a promising strategy in the search for better insect control.

## 1. Introduction

Mosquitos are the primary vectors of diseases such as malaria, yellow fever, dengue, chikungunya, West Nile, and Zika, which affect about 700 million and kill a million people each year. The World Health Organization (WHO) notes that more than half of the human population is currently at risk of mosquito-borne diseases. Moreover, there are concerns that progressive climate change may affect the extent of vector-borne disease outbreaks and insecticide sensitivity [1,2]. In 2019, malaria alone caused 229 million clinical episodes, which led to 409,000 deaths (WHO, World Malaria Report 2020). This primary life-threatening disease is transmitted through the bites of infected female *Anopheles* mosquitoes. 

Currently, the primary way to reduce malaria transmission and protect individuals is through the usage of repellents and/or insecticides. However, most mosquito species have become resistant to frequently used insecticides such as organophosphates, carbamates, and pyrethroids [3,4]. Regarding repellents, i.e., volatile substances that make mosquitos escape from a source of smell [5,6], the most effective broad-spectrum insect repellent is *N*,*N*-Diethyl-3-Methylbenzamide (DEET) [7]. Although it is considered safe, several reports in the last decades have indicated its potential adverse effects on human health and the environment [8,9,10,11,12]. Another active ingredient of repellents, 3-(*N*-*n*-butyl-*N*-acetyl)-amino-propionic acid ethyl ester (IR3535), is apparently safer for mammals. Furthermore, it is also known that DEET and IR3535 can elicit diverse modes of action [13,14,15,16,17], and can display novel non-classical effects, that can represent an alternative in the Insect Resistance Management for preventing the spread of mosquito-borne diseases. They can be used as a synergistic agent [18] to increase sensitivity to insecticides via a complex calcium-dependent intracellular signaling pathway following muscarinic acetylcholine receptor (mAChR) activation [19,20]. Searching for synergistic effects is becoming a popular strategy in the control of insect vector-borne diseases with a particular interest in the cholinergic system [21].

As acetylcholine (ACh) is the major signaling neurotransmitter in the insect nervous system, the ligand-gated ion channels named nicotinic acetylcholine receptors (nAChRs) are suitable targets for several classes of insecticides, including neonicotinoids, widely used in plant protection against insects since the late 1990s [22,23,24]. By contrast, mAChRs, which are G-protein-coupled receptors (GPCRs), are still unexploited in this area. However, recent studies have reported that insect mAChRs are interesting targets for repellents used as synergistic agents [18,19,20]. Both DEET and IR3535 were shown to interact with M1 andM3 mAChR subtypes at low concentrations inducing intracellular calcium rise that synergistically increase the insecticide efficacy when mixed with propoxur [19] and thiacloprid [20], respectively. This increased efficiency, based on the positive interaction between two compounds, represents a promising strategy to design more suitable approaches to control insect vector-borne diseases.

**Figure 1:**
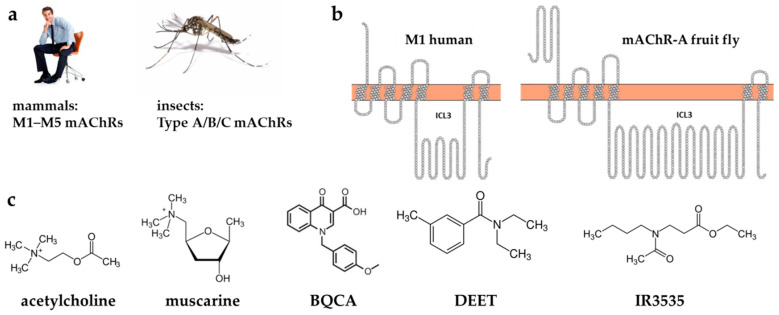
(**a**) Muscarinic acetylcholine receptors (mAChR) are divided into M1–M5 subtypes in mammals and A/B/C types in insects. (**b**) Snake plots [29] of human M1 (left) and fruit fly (*Drosophila melanogaster*) mAChR-A show the main structural features of a GPCR receptor—seven transmembrane helices linked by three extracellular and three intracellular loops among which the third one (ICL3) is the largest. Note that ICL3 is neither present in the human X-ray structure used in this study nor in the fruit fly homology model. (**c**) Structures of classical M1 agonists—acetylcholine, atropine; BQCA modulator and DEET and IR3535 repellents.

While mammalian mAChRs are divided into five subfamilies (M1–M5, Figure 1), three types have been identified in insects: type-A (mAChR-A), type-B (mAChR-B), and type-C (mAChR-C), and characterized in the fruit fly *Drosophila melanogaster* [25,26]. Very recently, mAChR-A have also been described in the malaria-spreading mosquito *Anopheles gambiae* [27]. The most closely homologous to the mammalian mAChRs is mAChR-A, which uses M1/M3/M5 types signaling pathways via activation of G_q/11_ protein and phospholipase C, resulting in the release of Ca^2+^ from internal stores through inositol 1,4,5-trisphosphate [28]. Like human types, it is activated by ACh and muscarine (see structures in Figure 1) and fully inhibited by classical mAChR antagonists such as atropine.

Using electropharmacological approaches together with the molecular docking of DEET and IR3535 to static human M1 and M3 receptor models, the ligand-receptor interactions and their mode of action were described [19,20]. However, the expected conformational changes in receptor structure with repellents bound could not be observed with this approach. Here, for the first time, we report on the molecular dynamics (MD) simulations of both insect and human mAChRs with repellent bound ligands.

Recent progress in the structure determination of GPCRs [30,31] triggered numerous studies in virtual screening and structure-based molecular design, which led to the development of new ligands for these receptors. To overcome the problem of limited subtype selectivity in small molecules targeting the highly conserved orthosteric sites (OS), there is a shift towards the allosteric ligands that bind in spatially distinct and less conserved sites (allosteric sites, AS) [32,33]. Allosteric ligands, apart from acting as the subtype-selective agonist or inverse-agonists, can modulate the efficacy and potency of orthosteric ligands. This effect is desirable because when they are used in new repellent formulations, it may allow for the reduction of doses of active substances. The discovery of bitopic (or dualsteric) ligands that occupy both binding pockets simultaneously [34,35,36] inspired researchers to fuse known orthosteric ligands with their allosteric modulators [37,38,39]. This approach, although challenging, may lead to the development of high-affinity subtype-selective molecules limiting the off-target activity and side effects of drugs. Therefore, here we propose a novel bitopic compound acting on insect mAChR. To assure a proper distance between the AS and OS ligands and to add a new dimension to chemical pest control, we augmented our design with a light-sensitive unit.

A promising approach that allows for the precise, reversible, and real-time spatiotemporal control of biological signaling is to use light as an external trigger to change a ligand shape and its pharmacological properties. A widely used molecular scaffold that changes structure upon irradiation is azobenzene [40,41]. Azobenzene has two isomers: the thermally stable trans and the thermally unstable cis isomer. Photoswitches, such as azobenzene, can be introduced into the structure of the bioactive ligands to control the activity of the target protein [41]. The mAChRs were the first GPCRs from the rhodopsin family that were successfully modulated by light upon binding of photoswitchable azobenzene-derived ligands [42]. Here we propose one prototype of hypothetically photoactive compound BQCA-azo-IR3535 and model its dynamical interactions with human and insect mAChRs.

The fact that the ligand binds to the receptor manifest in structural changes, often occurring in a place remote from the receptor site. Extensive analysis of all available experimental GPCR structures led to the discovery of a common and conserved activation pathway in class A GPCRs [43]. In this pathway, 34 residue pairs (formed by 35 residues, total) link a ligand-binding pocket with the G protein-coupling region of GPRCs via previously known structural motifs. These include a previously known set of four molecular switches: W6.48 tryptophan toggle switch (in CWxP motif), Y7.53 tyrosine toggle switch (in NPxxY motif), ionic lock involving helices TM3 and TM6 (D/ERY motif in TM3) and 3–7 lock linking helices TM3 and TM7 in the ligand-binding site [44] as well as a sodium ion-binding allosteric site D2.50 [45]. The pathway starts from the ligand-binding region and ends at the G protein interface going through four layers that reflect consecutive stages of the receptor activation.

In this study, we present, for the first time, a homology-based model of insect mAChR-A that may serve as a base for computational studies of repellents-GPCRs interactions. Based on the conserved activation pathway of class A GPCRs [43], we investigate the allosteric changes in mAChRs evoked by selected repellents and M1 modulators binding. All-atom MD simulations enable us to track the subtle, local alterations in the human and insect receptors conformations that accompany an early stage of their activation. Finally, we propose a novel type of bitopic and photoswitchable compound with a potential repellent activity and investigate its effect on mAChR-A dynamics.

## 2. Results and Discussion

We analyze two closely related molecular GPCR systems: human M1 and insect mAChR-A. The first structure is based on the X-ray data, and the second one is a homology-based model. One should remember that a direct comparison of results may be somewhat biased due to the hypothetical nature of the starting insect protein structure used for further MD modeling. However, due to the very good templates found for mAChR-A, this bias is acceptable. Reference human 150 ns MD GPCR data are used mainly to facilitate analysis and discussion for longer, 500 ns, insect mAChR-A trajectories.

### 2.1. Single Ligands Docking

#### 2.1.1. Single Ligands Docking to Human M1 mAChR

In the first step, we performed the molecular docking of eight ligands to the X-ray structure of human M1 mAChR (PDB code: 5CXV) using SMINA code [46], a fork for AutoDock Vina [47]. Ligands selected included repellents (DEET, IR3535), M1 agonists (muscarine, acetylcholine, oxotremorine-M), antagonists (atropine, pirenzepine), and modulators (BQCA, benzoquinazolinone 12). We have chosen the lowest energy poses of each ligand for further analysis. All ligands except pirenzepine and benzoquinazolinone 12 (bqz-12) docked with the best-scored poses in M1 OS, identified by FTSite [48] before docking. Pirenzepine docked well to both orthosteric and allosteric sites, while bqz-12 docked only to the AS. The poses of DEET and IR3535 are shown in the Supplementary Material (SM) Figure S1d,e. Interactions plots for all ligands made using the PLIP server [49] can be found in the SM Figures S2–S5.

DEET and IR3535 occupy the same orthosteric binding site as indicated by the residues (Tyr^3.33^, Tyr^7.38^, Tyr^6.51^, W^6.48^) that gave the highest contribution to the SMINA scoring function (SSF), presented in SM Figure S1b. The experimental alanine substitution of a majority of those residues strongly reduced the affinity of the endogenous agonist acetylcholine and the classical antagonist [50]. Superscripts refer to the Ballesteros–Weinstein numbering [51].

The SSF for DEET is equal to −7.3 kcal/mol. DEET in this pose is stabilized by pi-stacking interaction with Tyr381^6.51^, highly conserved in class A GPCRs. The highest contribution to the energy of binding gave interactions with the aromatic ring of DEET (the structure is shown in Figure 1c and the docking pose in SM Figure S1d). The binding affinity of IR3535 was slightly weaker with SSF = −6.26 kcal/mol.

#### 2.1.2. Single Ligands Docking to the Insect mAChR-A Model

**Figure 2:**
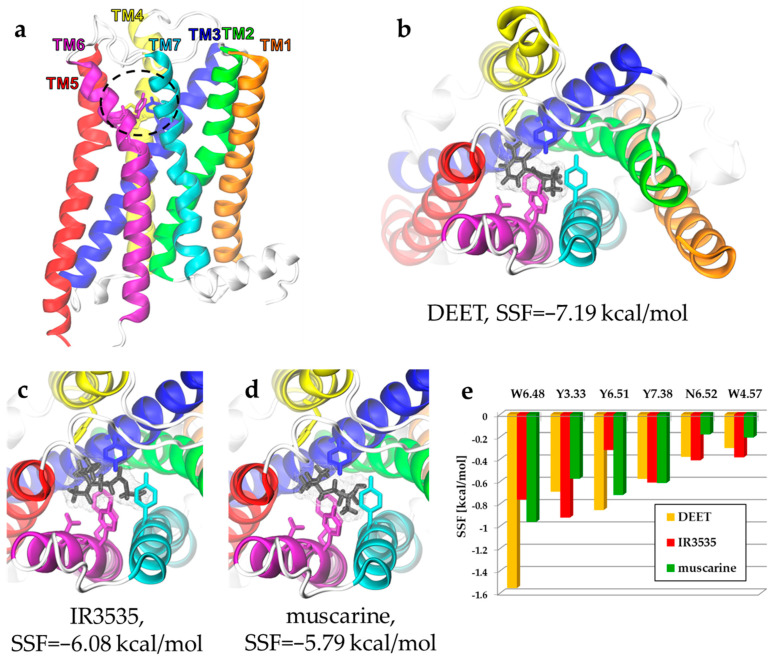
(**a**) The homology model of *Drosophila melanogaster* mAChR-A receptor based on the UniProtKB P16395 (ACM1_DROME) sequence and human M1 structure template (PDB code: 5CXV). The orthosteric binding-site region is marked with a black dashed line. (**b**–**e**) SMINA molecular docking of insect repellents DEET (**b**) and IR3535 (**c**) and classical agonist muscarine (**d**) to the homology model shown in (**a**). Top views are presented. (**e**) Docking energy decomposition presented as SMINA scoring function (SSF) in kcal/mol shows interacting ligand residues of the mAChR-A orthosteric binding site.

There are no experimental structures for any type of insect muscarinic receptors. To check how the effects of ligands binding manifest in insect type-A receptors, we build the homology model of a fruit fly (*Drosophila melanogaster*) mAChR-A protein (Figure 2a). As the cytoplasmic loop connecting TM5 and TM6 helices (ILC3) is very long (431 residues, more than a half of the whole receptor sequence length, see Figure 1b), the model could be built only after the removal of this intracellular part. Such simplification in GPCR structure modeling is a typical procedure. The dockings of DEET, IR3535, and muscarine were performed using the same protocol as for the human M1 described in the methods section.

In insect mAChR-A, the best docking score (i.e., low SFF value) was observed for DEET (−7.19 kcal/mol), followed by IR3535 (−6.08 kcal/mol). According to our approach, muscarine binds weaker to_mAChR-A (SFF = −5.79 kcal/mol). The order of these numbers is the same for the human M1 GPCR, but the binding affinity is predicted to be slightly weaker (ΔSFF = 0.1–0.2 kcal/mol). Similar to M1, in the insect receptor, the strongest stabilization of ligands comes from aromatic residues (five out of six presented in Figure 2e). The lists of most stabilizing residues in both receptors are identical (cf. Figure 2e and Figure S1b). Clearly, the tryptophan toggle switch (Trp^6.48^) plays a major role in the OS of both receptors. This strong similarity in repellents’ affinity to insect and human receptors may indicate possible physiological effects of DEET in humans, induced via the M1 activation pathway. The docking is only a computational procedure, sensitive to numerous parameters, including details of the hypothetical docking site geometry. However, it is an interesting observation that IR3535 shows weaker binding than DEET, so its negative effect is expected to be somewhat weaker.

### 2.2. What Happens upon Ligand Biding? Dynamical Response of Human M1 Receptor

Ligands present in the orthosteric (or allosteric) site exert their biological roles by changing the conformation of GPCRs. The transduction of those signals to remote places such as the G-protein binding site requires mechanical interactions occurring within the receptor body. Tightly packed helices, present in the intramembrane part, facilitate this task but not all out of hundreds (>800) possible residue–residue contacts are critical for such signal transduction. The role of such contacts in M1 and mAChR-A has not been investigated thus far. The whole process of triggering a response to a ligand is too long for computational investigation, but the important initial stages of signal transduction may be monitored using MD simulations on hundreds of ns timescale. Thus, to get a dynamical picture of repellents induced changes in GPCRs, we performed MD simulations and analyzed closest contacts in regions delineated in extensive studies by Zhou et al. of all available X-ray type-A GPCR structures [43].

In the first step, the lowest energy docking poses were used as starting points for the MD simulations of the M1 receptor embedded in a lipid bilayer. We performed three repetitions of 150 ns long MD for M1 receptor without ligand (APO) with DEET, IR3535, and muscarine. RMSD plots indicate (data not shown) that reasonable convergence has been achieved in our relatively short simulations.

On a relatively short timescale exploited here, the analysis of contacts with the GetContacts server [52] or RRCS contact scores using a method introduced recently [43] may be illuminating.

RRCS is an atomic distance-based parameter that quantifies the strength of contact between residue pairs by summing up all possible inter-residue heavy atom pairs without weighting factors [43]. Not only does it capture side-chain repacking if the backbone atoms of the two residues are close to each other, but it also describes local contacts involving adjacent residues (excluding backbone atoms of residues that are within four amino acids in protein sequence). Thus, RRCS can be used as a quantitative descriptor of dynamical contact rearrangement in protein and a useful tool for the comparison of multiple receptor states. Zou et al. performed RRCS calculations on all available high-resolution 3D structures of class A GPCRs comparing their active and inactive states. The universal signal transduction path has been proposed consisting of 34 pairs of AA [43]. Unfortunately, it is not known how such RRCS values evolve over time, how they change upon ligand-binding, and to what extent the indicated critical contacts last.

#### 2.2.1. GetContacts

**Figure 3:**
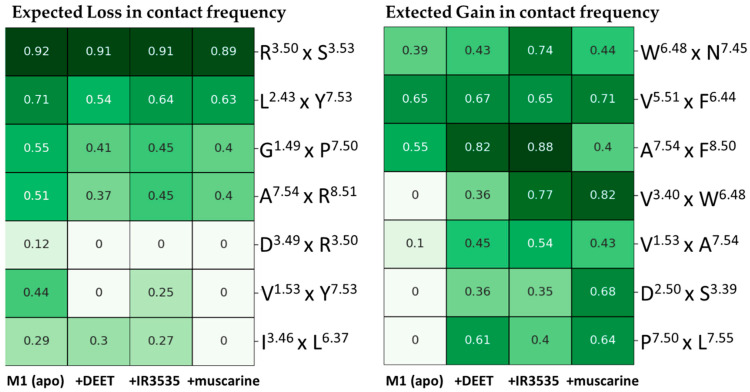
Average contacts as a fraction of total simulation time (450 ns) [52] observed for M1 apo and for M1 having DEET, IR3535, and muscarine ligands in the orthosteric site.

To examine the initial response of the M1 receptor to the ligands, we calculated fractions of simulation frames in which a given residue pair, found in [43] as a part of the signal transduction pathway, form a contact. In Zhou et al. meta-analysis [43], two types of structural response upon ligand-binding were postulated: (i) some residue residue pairs increase their contact frequency in the activated form of GPCR with respect to the apo or inactivated protein, and (ii) other pairs decrease contacts frequency. Initially, we used for the analysis GetContacts server [52]. Here, Van der Waals contacts (vdw) between two atoms are registered if the distance between their centers is less than the sum of their van der Waals radii plus an epsilon value of 0.25 Å. Data for representative 14 pairs (out of 34 indicated by Zhou et al.) that exhibited the most profound variations between molecular systems are shown in Figure 3. Contacts between other residues pairs from the pathway set of Zhou et al. [43] were not affected by the presence/absence of our ligands.

Such a dynamic picture is very interesting. Indeed, all pairs which were predicted to decrease contacts upon agonist binding show a decrease in our MD calculated contact fraction (see Figure 3, left panel) and vice-versa: all residue pairs that should increase their contacts when the ligand comes into the orthosteric site do have substantially higher contact fraction (Figure 3, right panel). We estimated the global effect (GE) of ligand-binding using the following metric:

$$\mathrm{GE}+=\sum_{i=1}^{7}{\left(\mathrm{ACF}{\left(+\right)}_{i}^{\mathrm{APO}}-\;\mathrm{ACF}{\left(+\right)}_{i}^{\mathrm{LIG}}\right)}^{2}$$

$$\mathrm{GE}-=\sum_{i=1}^{7}{\left(\mathrm{ACF}{\left(-\right)}_{i}^{\mathrm{APO}}-\;\mathrm{ACF}{\left(-\right)}_{i}^{\mathrm{LIG}}\right)}^{2},$$

where ACF(+/−) are averaged contact fractions (see Figure 3) for gain and loss in contact frequency, respectively, LIG = DEET, IR3535, or muscarine.

From Table 1, one can see that DEET exerts its effect on M1 GPCR in a similar way as muscarine, but the decrease of frequency contacts (GE− = 0.829) is much weaker than that for muscarine (DE− = 1.682). IR3535 basically does not increase the contacts between critical pairs but strongly reduces that number for another set of residues, its GE− of 1.30 is close to GE− calculated for muscarine.

We found that for our three agonists, in general, the highest gain in contact frequency in human M1 was observed between the tryptophan toggle switch Trp378^6.48^ with Val113^3.40^, the residue contributing to the sodium ion binding pocket Asp71^2.50^ with Ser112^3.39^ and between residues located on the H7 Pro415^7.50^ and Lys420^7.55^. Loss of contacts was noted in highly conserved DRY motif Asp122^3.49^ × Arg123^3.50^, Val46^1.53^ with the tyrosine toggle switch Tyr418^7.53^ and in interactions of I119^3.46^ with the microswitch Lys367^6.37^.

#### 2.2.2. Analysis of Residue-Residue Contact Scores in Human M1

The recently proposed [43] residue–residue contact scores (RRCSs) are better suited for the analysis of the subtle effects in GPCR structure induced by ligands than the simple contact fraction presented in the previous section. RRCS takes into account further located atoms and is defined as:

$$\mathrm{RRCS}=\sum_{i\in A}\sum_{j\in B}\delta_{\mathrm{ij}}$$

where,

$$\delta_{\mathrm{ij}}=\{\begin{array}{c} 1\;r_{\mathrm{ij}}\leq r_{\min},\;\\ 0\;r_{\mathrm{ij}}\geq r_{\max},\\ ({\left(r_{\max}-r_{\min}\right)}^{-1}\left(r_{\max}-r_{\mathrm{ij}}\right)\;\mathrm{otherwise}, \end{array}$$

and r_ij_ is the distance between i and j-th atom, and r_min_ = 3.23 Å and r_max_ = 4.63 Å. In our case, group A contains atoms of one residue of the investigated pair and group B consists of atoms belonging to the other residue in the analyzed pair.

**Figure 4:**
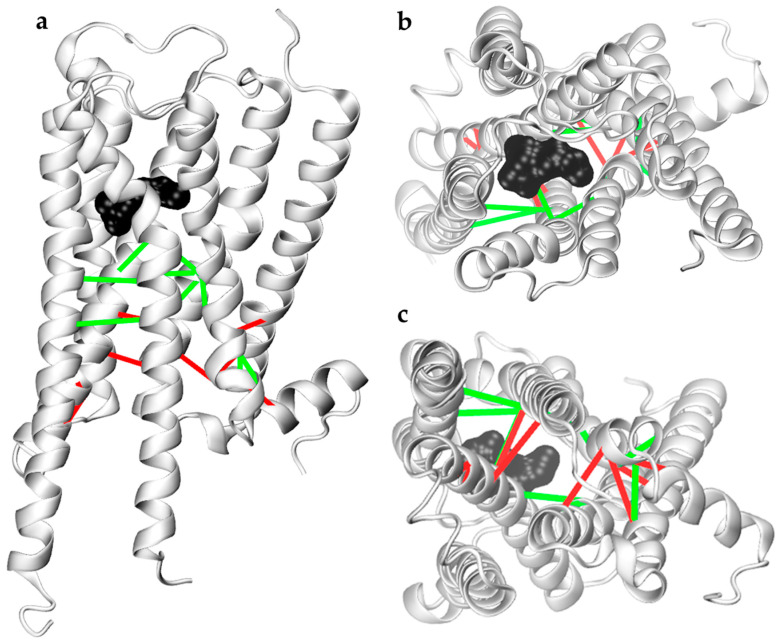
Contacts between key residue pairs involved in the first stage of M1 receptor activation discussed in this paper. (**a**) Side, (**b**) top, and (**c**) bottom views of M1 are shown with distances that increase (green) and decrease (red) upon activation, represented by lines. IR3535 is shown in black surface representation to indicate the position of the orthosteric binding site.

We applied this approach to investigate differences in contacts between all 34 key residue pairs of the M1 receptor caused by agonist muscarine and repellents DEET and IR3535. Half of the 34 residue pairs contacts were not affected by interactions with ligands. Therefore, we limited the further analysis to nine pairs that increased contacts upon muscarine binding (Figure 4, green lines) and eight pairs that decreased (or loose) contacts while ligand-bound (Figure 4, red lines). Among them, 5 were intrahelical and 12 were interhelical.

**Figure 5:**
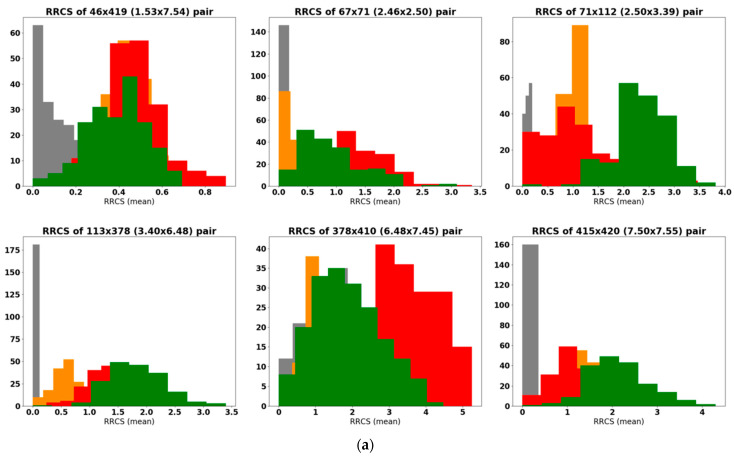

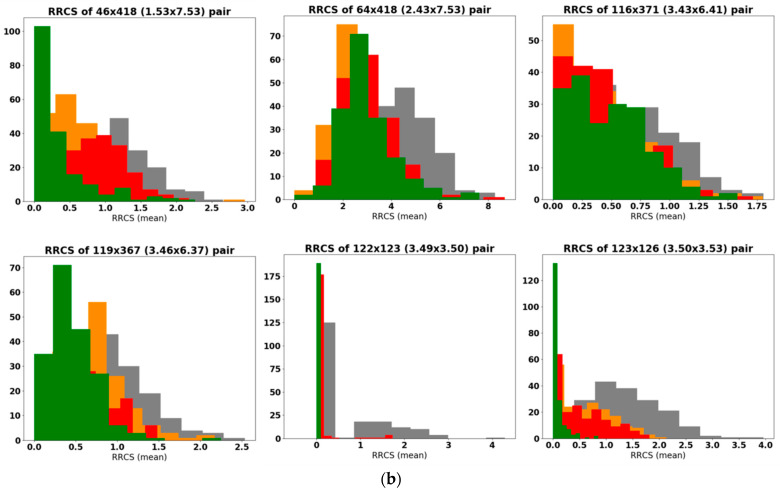
(**a**,**b**) Histograms of residue–residue contact scores (RRCSs). Sampling was 1 frame/0.8 ns of 150 ns MD simulation (average of 3 repetitions). Contacts that increase (**a**) and decrease (**b**) RRCS are shown for M1 apo receptor in grey, M1 with muscarine in green, M1 with DEET in orange, and M1 with IR3535 in red.

In Figure 5a,b we show RRCS histogram plots for the bare M1 receptor (apo, grey) and M1 with a ligand in its orthosteric site: muscarine (green), DEET (orange), and IR3535 (red). Figure 5a presents the contacts that were strengthened during activation, while in Figure 5b, the residue pairs that loosened contact during activation are shown. Histograms of the RRCSs for other interesting but less affected RRCS are presented in the SM Figure S6. The effect of muscarine is clearly seen as this ligand moved the mean RRCS values of M1 residue pairs towards the active form of GPCR (residue pairs from Figure 5a are much closer than in the apo form, while those in Figure 5b have loosened their short distances). We found that the effect of repellents was located somewhat in between APO and muscarine, and IR3535 increases contacts to a larger extent than DEET does. Notably, the indicated residues corresponded to the known, highly conserved classical points of GPCR activity regulation: the residue contributing to the sodium ion binding pocket Asp71^2.50^, the hydrophobic lock Leu116^3.43^, Asp122^3.49^, and Arg123^3.50^ from the DRY motif, the microswitch Leu367^6.37^, the tryptophan toggle switch Trp378^6.48^, and the tyrosine toggle switch Tyr418^7.53^ [44].

The shapes of the RRCS histograms allow for the qualitative assessment of the mobility of a given amino acid pair: the wider the distribution, the more flexible the region. In Figure 5a, we see a strong impact of the ligands on Val113^3.40^ × Trp378^6.48^ and Pro415^7.50^ × Leu420^7.55^ pairs, since, in M1 apo, there was no contact, but in the ligands’ activated forms, such contacts were formed. Notably, the most important pairs involved in the GPCR activation process determined by the GetContacts server [52] were also independently discovered in our more precise RRCS analysis.

#### 2.2.3. In Search for Repellent Modulation: Sequential Docking and Dynamics of the Human M1

**Figure 6:**
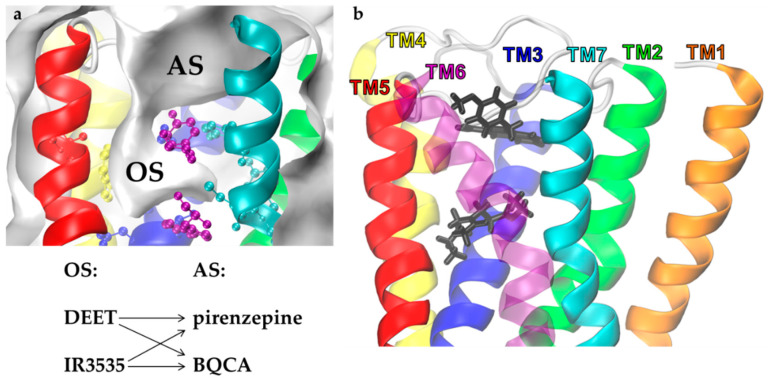
Multiple molecular docking of insect repellents and modulators to the human M1 muscarinic receptor. (**a**) Surface representation of orthosteric (OS) and allosteric (AS) sites of M1 receptor and scheme of multiple docking protocol. (**b**) Docking poses of IR3535 in the OS (bottom, black) and BQCA in the AS (top, black); the key residues of OS are marked.

In M1 GPCR, both OS and AS may be occupied by small ligands at the same time. We expect that an extra ligand in AS may enhance selectivity and the action of repellents by a positive allosteric modulation. To develop a bitopic ligand with a repellent function that could occupy both sites simultaneously, we examined how the presence of allosteric modulators influences the effect of repellents on the receptor structure. We docked pirenzepine to the M1 with DEET or IR3535 in the orthosteric site of M1 (Figure 6). The same study was repeated for the modulator/agonist BQCA. Those four systems dynamics were simulated (150 ns × 3 repetitions for each system) to monitor RRCSs structural parameters and to compare with the single ligand cases.

We analyzed all pairs from the consensus signal transduction pathway in which RRCS were affected by DEET or IR3535 (see Figure 5a,b and Figure S7a,b), looking for the modulatory effect of allosteric ligands. The presence of both pirenzepine and BQCA reduced the impact of DEET on RRCS values (data not shown). We expected that those modulators would not potentiate DEET repellent activity.

**Figure 7:**
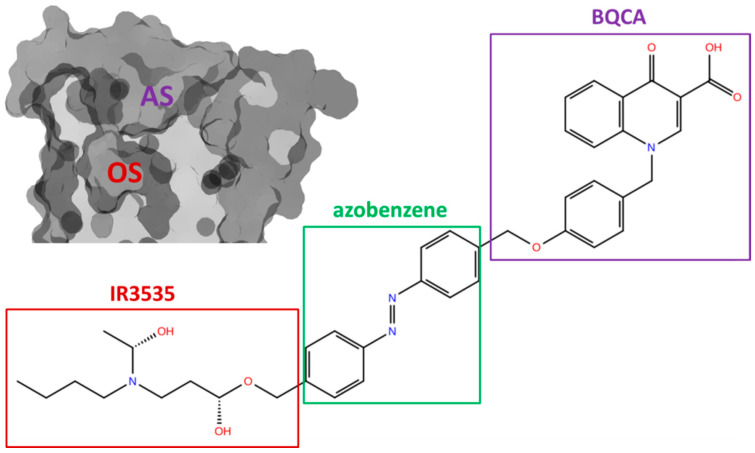
Structure of the bitopic, photoswitchable insect repellent BQCA-azo-IR3535 with parts that target orthosteric (OS, red) and allosteric (AS, violet) sites of the muscarinic acetylcholine receptor (left side) indicated in red and purple boxes, respectively.

More promising results were found for IR3535. While pirenzepine slightly and negatively modulated the action of IR3535, we observed symptoms of positive modulation of IR3535 impact on M1 by BQCA docked to AS (results of RRCSs from MD are presented in SM Figure S7a,b as reference data and are discussed further). Based on this observation, we proposed a new, possibly photoswitchable compound composed of BQCA, a linker, and IR3535 (BQCA-azo-IR3535). As a linker, we applied azobenzene since it has a proper size and useful photophysical properties. The structure of this test molecule is shown in Figure 7.

### 2.3. Computer Modeling of Designed Bitopic Ligand (BQCA-azo-IR3535)

Bitopic ligands designed for better modulation of the mAChRs were studied in the past. For example, the M2 agonist Phthalimide-Azo-Iperoxo, which links the fragments of muscarinic agonist iperoxo and allosteric modulator W84 via azobenzene functional group, was proposed recently by Riefolo et al. [53]. Azobenzene in its ground state was extended, i.e., trans conformation. It activated the M2 receptors and can be reversibly photoswitched to enable precise spatiotemporal control of cardiac function [53]. Another bitopic ligand—BQCAAI—was obtained by connecting the agonist iperoxo with the positive allosteric modulator BQCA through an azobenzene linker as well. Strikingly, cis-BQCAAI acts as an antagonist (under 366 nm) of the M1 receptor, while trans-BQCAAI is an agonist (under dark conditions or 455 nm illumination) [39]. These results inspired us to search for a similar system with repellents as main part ligands. Using a molecular builder (see Methods), we drafted a skeleton and optimized the geometry of the BQCA-azo-IR3535 derivative (see Figure 7).

#### 2.3.1. Bitopic BQCA-azo-IR3535 Ligand Effect on Human M1 GPCR

As expected, SMINA molecular docking of IR353-azo-BQCA to human M1 receptor showed that the IR3535 part occupies the orthosteric site, while the modulator part (BQCA) was docked in the allosteric site (see a comparison of IR353-azo-BQCA docking poses in human and insect receptor in SM Figure S8). The trans-azobenzene linker fits into a narrow grove connecting the pockets. The binding energy (SSF) was equal to −11.35 kcal/mol, while for IR3535 alone, SSF was −6.26 kcal/mol and for BQCA −8.6 kcal/mol. Thus, our bitopic ligand should have a higher affinity towards M1 than any of these two ligands.

The lowest energy pose of BQCA-azo-IR3535 was used for MD simulations (3 × 150 ns) of the ligated M1 receptor. The comparison of RRCS values for an M1 receptor with IR3535 alone and IR3535 together with unlinked BQCA and the receptor with BQCA-azo-IR3535 are shown in the histogram plots in SM Figure S7a,b.

Our large, bitopic ligand affects contacts between critical residues of human M1 in a way that is not a simple superposition of BQCA and IR3535 ligands effects (SM Figure S7a,b). In general, we do not observe the strengthening of contacts in the receptor with BQCA-azo-IR3535 (in comparison to the APO form), but rather the loosening of M1 packing is seen. In the RRCSs analyzed here, the contacts of helix H6 are present six times. This helix is particularly important since it made an outward movement upon GPCR activation. The bitopic ligand exerted no modulatory effect in tightening contacts on those pairs. However, a strong loosening of H6 (Leu116^3.43^ × Leu371^6.43^ and Ile119^3.46^ × Leu367^6.37^) induced by this ligand was observed. Of special interest is the 116^3.43^ × 371^6.41^ pair, which, together with the 116^3.43^ × 370^6.40^, is known as the hydrophobic lock [44]. This region was loosened as 116^3.43^ × 371^6.41^ contacts decreased to zero in 1/3 of BQCA-azo-IR3535 simulation frames. Such reduction is even stronger than that induced by muscarine (Figure 5b). The effect of BQCA-azo-IR3535 on the Ile119^3.46^ × Leu367^6.37^ microswitch pair was comparable to that exerted by muscarine.

The MD results are encouraging in the sense that the good quality M1 structure was not affected much, and a strong affinity for the bitopic ligand was predicted. Now, we have a good reference point for more extensive MD studies of bitopic ligand action in insect mAChR-A.

#### 2.3.2. Bitopic BQCA-azo-IR3535 Ligand and Insect mAChR-A Dynamics

**Figure 8:**
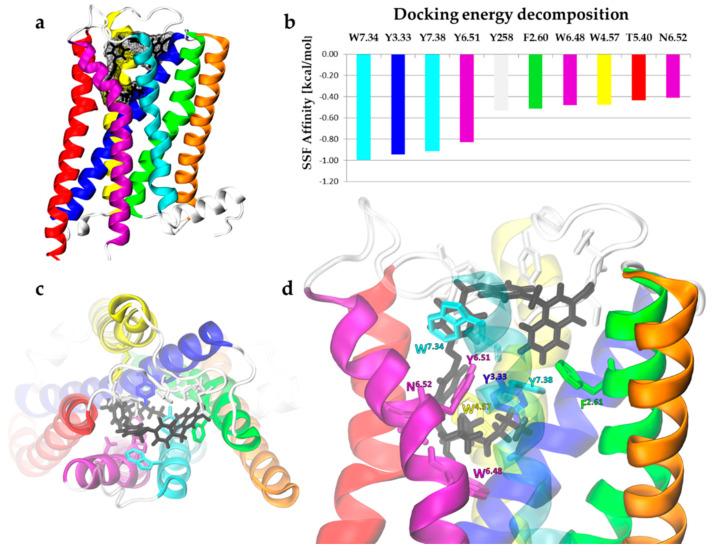
Molecular docking of bitopic ligand BQCA-azo-IR3535 to the homology model of *Drosophila melanogaster* mAChR-A. (**a**) Receptor structure with the ligand-bound (black licorice and surface representation). Note that intracellular loop 2 was removed from the model. (**b**) Docking energy decomposition shows residues giving the highest contribution to the affinity of ligand-binding. These residues are marked in (**d**). The top view (**c**) and the side view (**d**) of the BQCA-azo-IR3535 (black) docked to the mAChR-A.

We docked BQCA-azo-IR3535 ligand to human and insect GPCR models using the same methodology (Figure 8). Similar to the M1 receptor in mAChR-A, the BQCA part went to AS, and IR3535 part fit well into the orthosteric cavity. The total SSF value for the bitopic ligand-binding to the insect receptor equals −11.97 kcal/mol (while −11.35 kcal/mol was obtained for the human M1). The docking energy decomposition showed that the highest contributions to the binding of BQCA-azo-IR3535 again produced the aromatic residues (Figure 8b). Most of the binding energy came from the interaction of the repellent part with the OS. Only three of these residues (W^7.34^, Y258 from the extracellular loop 2, and F^2.60^) contributed to the BQCA part binding in the AS, while the T5.40 interacted with the azobenzene linker. The comparison of BQCA-azo-IR3535 docking poses to the aligned M1 human and insect mAChR-A structures, together with the energy decomposition, are provided in SM Figure S8. To investigate the differences between the repellents binding to the active state receptors, we performed docking of the DEET, IR3535, and BQCA-azo-IR3535 to the X-ray structure of human M1 in its active state (PDB code: 6OIJ) to the insect mAChR-A model built using this template, and also to the most recently released human M1 structure (PDB code: 6ZG9). SSF values can be found in the SM Table S1.

To assess the action of the BQCA-azo-IR3535 ligand on insect receptor dynamics, we performed longer MD simulations of mAChR-A without a ligand (APO) and with IR3535, muscarine, and bitopic ligands. Three independent 500 ns simulations were run for each system (note that the simulation time was over three times longer than for the human M1 receptor, so conformational space for the less reliable model is well sampled).

The protocol used was the same as for the M1 receptor (see Methods) with the plasma membrane composition modification to obtain more insect-like lipid content.

The plasma membrane in which receptors are embedded provided not only a neutral environment but also affected the ligand affinity [54]. The majority of MD simulations of membrane proteins had been performed assuming in human-like membrane models (usually phosphatidylcholine: POPC or DOPC). However, flies differ in their lipid composition from humans substantially. Insects have an inverted and four times higher phosphatidylethanolamine to phosphatidylcholine ratio than mammals [55]. Thus, using CHARMM-GUI [56], we created a heterogeneous bilayer model composed of: 38% DOPE, 18% DOPS, 16% DOPC, 13% POPI, 11% SM (CER180), 3% DOPG and 1% PALO 16:1 fatty acid.

**Figure 9:**
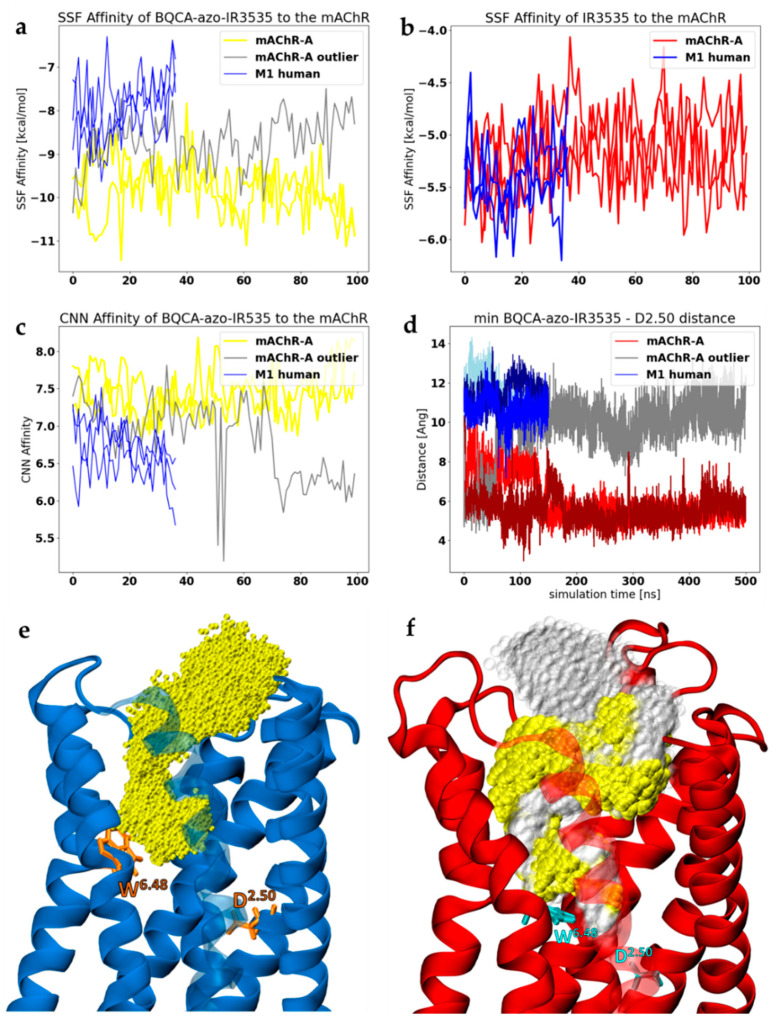
BQCA-azo-IR3535 interaction with mAChRs. The SSF affinity evolution for bitopic ligand (**a**) and IR3535 (**b**) are shown for mAChR-A and, for the reference, for the human M1 receptor. (**c**) The convolutional neural network (CNN) scoring function, measured in “pK” units where 1 μM is 6, 1 nM is 9, is plotted for BQCA-azo-IR3535 interaction with mAChRs. (**d**) The distance [Å] between the closest non-hydrogen atom of a ligand and the D^2.50^ residue (sodium pocket) of human M1 (blue) and insect mAChR-A (red and grey). (**e**,**f**) Positions occupied by non-hydrogen atoms of BQCA-azo-IR3535 (yellow) in the human M1 receptor (**e**) and insect mAChR-A model (**f**). The W^6.48^ and D^2.50^ residues are marked (orange in human and cyan in insect mAChR) as indicators of the distance between ligand OS and the sodium ion-binding site. For all plots, data collected from the 3 × 500 ns MD simulation for the human M1 receptor and 3 × 500 ns for the insect mAChR-A are shown.

Data from the MD showed that BQCA-azo-IR3535 was more tightly bound to the insect receptor than to the human one (Figure 9). In Figure 9a, we present dynamical changes in values of SSF, which are proportional to binding affinity, for numerous MD structures “on-the-fly”. Except for one ”outlier” trajectory (grey in Figure 9a,c,d), we observed that the SFF for the bitopic ligand in insect GPCR for the first 150 ns is systematically lower (Figure 9a) than that calculated for the human M1. We extended our simulations for the insect mAChR-A, and, indeed, the good SFF values were kept low throughout the whole simulation. The same analysis performed for the IR3535 ligand showed no differences between those species (Figure 9b). This conclusion is supported by the convolutional neural network (CNN) data (Figure 9c). The CNN scoring function, measured in “pK” units, may be easily converted to the ligand affinity, where 1 μM is 6, and 1 nM is 9, so the higher CNN, the better. Thus, affinity towards the humans calculated using GNINA software [57] was around 1 μM, and towards the insects, close to 10 nM. We inferred that the bitopic ligand should have a stronger physiological effect in insects than in humans.

**Figure 10:**
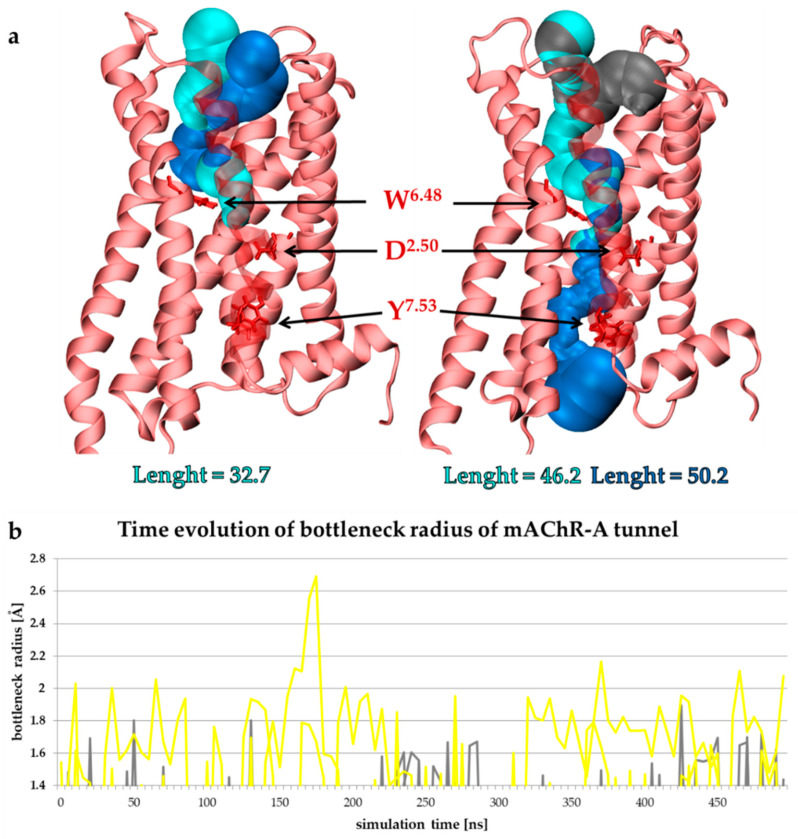
(**a**) Tunnels were found in insect mAChR-A receptor at the minimized and equilibrated structure (left) and in the representative MD snapshot of the receptor with BQCA-azo-IR3535 ligand. (**b**) The bottleneck radius of the tunnels created below the ligand-binding site allows water flow (i.e., with the bottleneck radius higher than 1.4 Å). In gray, the tunnels found in the 3 × 500 ns MD of the APO mAChR-A are shown, while in yellow, those found in the receptor with the bound BQCA-azo-IR3535. Tunnels were visualized using MOLE 2.0 [62], and the bottleneck analysis was performed with CAVER [63].

The space occupied by the bitopic ligand in both receptors was very large. The overlaps of all positions occupied by non-hydrogen atoms of BQCA-azo-IR3535 (yellow) in M1 (blue) and mAChR-A (red) receptor during three repetitions of MD trajectories are shown in Figure 9e,f, respectively. The ligand was bound deeper in mAChR-A, and moved towards the sodium ion pocket identified as a highly conserved D^2.50^ residue (Figure 9f and Figure 10a). Note that one mAChR-A trajectory (shown in gray in Figure 9f) is perhaps an outlier: in the last 30% of the 500 ns trajectory, the ligand shows a tendency to leave the allosteric pocket. The evolution of the distance between the ligand and the D^2.50^ residue is shown in Figure 9d. The small and flexible endogenous agonist acetylcholine was found to be able to diffuse from the OS into the new binding site next to the D^2.50^ residue of M3 and M4 muscarinic receptors [58]. The sodium ion located at D^2.50^ was present in inactive conformations of most GPCRs, but not in agonist-bound ones. As a negative allosteric modulator of receptor activation, it stabilized the inactive state of the receptor, decreased affinities for agonists, and enhanced affinities for some antagonists [59]. A strong sensitivity to the sodium ion has been shown for a negative allosteric modulator SB269652 that adopted an extended bitopic pose in the dopamine D2 receptor and completely lost its modulatory effect in the absence of sodium ion [60]. In turn, BMS986122, a positive allosteric modulator of the µ-opioid receptor, was found to exert its effect through disruption of the sodium binding, thereby promoting receptor activation [61]. We speculate that a similar effect may happen for the BQCA-azo-IR3535 ligand-bound into mAChRs.

Water plays an important role in GPRC-mediated signaling. We investigated the formation of the tunnels in mAChR-A. (Figure 10). While the hydrophobic layer was present at t = 0 and water cannot flow through the receptor (Figure 10a, left panel), the presence of BQCA-azo-IR3535 ligand promoted the formation of a tunnel between the OS and the G-protein binding site (dark blue in Figure 10a, right panel). The bottleneck was formed by a tryptophan toggle switch W^6.48^ (part of the CWxP motif). The disruption of water-mediated interactions between W^6.48^ and D^2.50^ was critical for the receptor activation, as the incoming water that passed the W^6.48^ gate changed the conformation of the tyrosine toggle switch Y^7.53^ (part of the NPxxY motif) to allow a continuous pathway of water molecules to the cytoplasm [44]. We investigated the dynamic changes of the bottleneck radius in 3 × 500 ns MD trajectories of mAChR-A with no ligand (APO, grey in Figure 10b) and with the bitopic-bound ligand (yellow in Figure 10b). The instances of bottleneck radius value >1.4 Å are shown to illustrate the fraction of MD in which the water flow is allowed. The difference between the APO and ligand-bound receptor conformation is evident.

**Figure 11:**
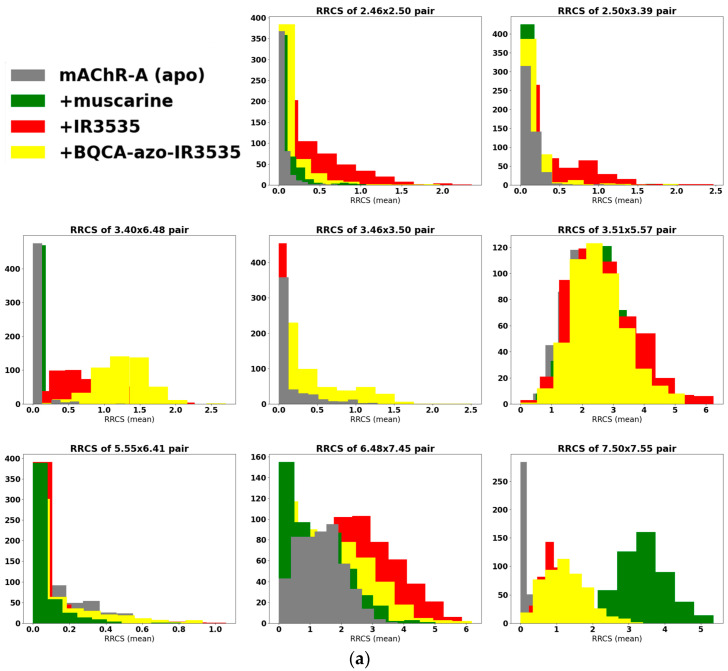

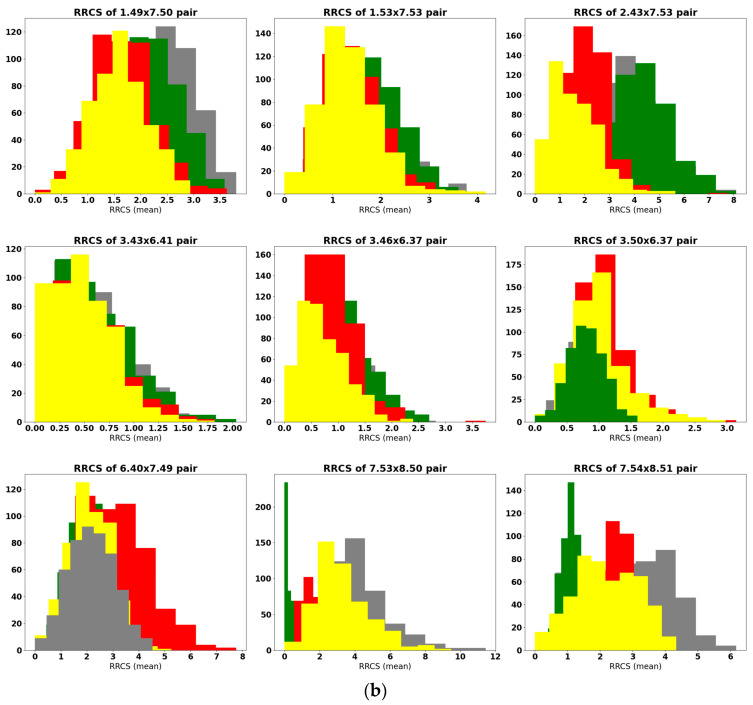
(**a**,**b**) Histograms of residue–residue contact scores (RRCSs) calculated for 1 frame/1 ns of 500 ns MD simulation (average of 3 trajectories) of insect mAChR-A Apo receptor (in grey). Contacts that increase (**a**) and decrease (**b**) RRCS upon activation by a ligand are shown for muscarine in green, IR3535 in red, and for BQCA-azo-IR3535 in yellow.

Finally, we return to the RRCS analysis. Data for 500 ns long simulations for the insect APO mAChR-A and the receptor with muscarine, IR3535, and BQCA-azo-IR3535 ligands are presented in Figure 11a,b. In a few cases, significant changes in the RRCS patterns induced by ligands were observed. The most striking effect we saw was for the Val^3.40^ × Trp^6.48^ pair that corresponded to the tryptophan toggle switch: the bitopic ligand dramatically increased RRCS with respect to APO and muscarine forms.

A comparison of data presented in Figure S7a,b and Figure 11a,b may reveal possible differences in structural effects exerted by our bitopic ligand on human and insect receptors, respectively. Similar to human M1 GPRC, the bitopic ligand does not strikingly strengthen the mAChR-A contacts in the signaling pathway pairs (Figure 11a).

However, bitopic ligand decreases the contacts of some critical pairs in the insect receptor structure stronger than IR3535 or even stronger than muscarine (Figure 11b). Particularly interesting were the contacts involving the tyrosine toggle switch Tyr^7.53^ (NPxxY motif): Val^1.53^ × Tyr^7.53^ and Leu^2.43^ × Tyr^7.53^. We recall that substantial dynamical changes of the NPxxY motif are typically observed when the GPCR is bound to its full agonist [64]. Tyr^7.53^ was found to switch between three rotameric conformations affecting water flow through the receptor. Upon GPCR activation, a hydrophobic layer breaks as a continuous water channel is formed from the ligand-binding pocket to the cytoplasm [64]. This postulate is in accordance with our analysis of the water tunnel formation (Figure 10) in mAChR-A with BQCA-azo-IR3535.

Quite interesting to note is the behavior of the Gly^1.49^ × Pro^7.50^ pair: in human M1, the large BQCA-azo-IR3535 ligand makes this contact stronger, while in insect mAChR-A those residues are pushed away, and contacts are weaker.

In our detailed, molecular-level analysis, we indicated sensitive spots in the muscarinic GPCRs activation pathway. The characterization of early-stage conformational changes in both human and insect receptors in response to repellent ligand-binding provides ground for the development of new chemicals that would be selective towards insects. Our in silico analysis of a novel, bitopic, and photoswitchable ligand BQCA-azo-IR3535 interactions with mAChRs calls for further experimental studies.

## 3. Conclusions

MD simulations, despite all known limitations related to simplified models when experimental structures are scarce and limited sampling, are widely used in studies of membrane proteins, including the conformational changes of GPCRs induced by ligands [65,66]. Even though the timescale of GPCR activation by agonists is too long for classical MD, the dynamics of microswitches revealed that relatively short simulations could indicate important allosteric coupling [67]. 

In this work, for the first time, we provided molecular insights into the early-stage responses of human and insect muscarinic receptors on activation by the safe repellent IR3535 and its bitopic, photoswitchable derivative BQCA-azo-IR3535. The concept of this ligand was based on the positive allosteric modulation of IR3535 by BQCA we observed in the human M1 receptor. IR3535 was linked to the positive allosteric modulator BQCA part that binds receptor mAChR in the less conserved AS. The ligand proposed here is a molecule that may, hopefully, combine repellent activity with selectivity.

Based on the docking of ligands to the human M1 X-Ray structure and our homology model of the *Drosophila melanogaster* mAChR-A GPCRs and MD simulations, we analyzed the dynamical responses of receptors to the repellents. The recently proposed signal transduction pathway for class A GPCR [43] enumerated 34 pairs critical for receptor activity. After careful analysis of the differences in the close contacts of the RRCS parameters calculated from the 3 × 500 ns MD data sets, we identified pathway pairs that were affected substantially by ligand-binding. The most profound structural effects were localized in the following regions: (a) the tryptophan toggle switch Trp^6.48^ with Val^3.40^, and (b) the residues located at H7 Pro^7.50^ and Lys^7.55^. Substantial loss of contacts was noted in (c) Lys^2.43^ with the tyrosine toggle switch Tyr^7.53^ and (d) in interactions of Ile^3.46^ with the microswitch Lys^6.37^. The MD simulations analysis suggests that the large, bitopic ligand BQCA-azo-IR3535 was bound more tightly to the insect mAChR-A than to the human M1 and, therefore, may increase the rate at which the insect GPCR transition to the active conformational state more profoundly. Thus, based on the presented limited modeling, we believe that the strategy of using simultaneous modulators of both orthosteric and allosteric sites in pest control studies is promising. Such investigations, especially aimed at GPCRs [68], could bring new compounds with reduced toxicity to humans.

## 4. Materials and Methods

### 4.1. Molecular Docking

3D structures of the ligands were downloaded from PubChem [69] and docked to the inactive structure of the whole M1 receptor protein (PDB code: 5CXV) using SMINA package [46], a fork of Autodock Vina [47] that provides enhanced support for minimization and scoring. For each ligand, 10 independent docking runs were carried out using default settings, generating up to 100 poses per run. The best-scored poses of the ligands occupying the pockets found using the FTSite [48] and POCASA1.1 [70] were selected and further prepared using Schrödinger Maestro [71] by adding hydrogen and were minimized to obtain optimal conformation.

### 4.2. Molecular Dynamics (MD)

Topology and parameters files for the ligands were generated by SwissParam [72]. The proper orientation of the receptor in a membrane was found using the PPM OPM server [73]. Each human receptor with a ligand system was placed in a homogenous lipid bilayer environment consisting of approximately 200 (190–207) dioleoylphosphatidylcholine (DOPC) molecules. About 21,000 (20,968) molecules of water were added above and below the lipids to generate a 20 Å thickness layer. The system was neutralized with counterions to the concentration of 0.15 M. Temperature was controlled by the Langevin thermostat with a value of 303.15 K and the target pressure was set to 1.01325 bar (1 atm). We applied the CHARMM36 force field with the TIP3P model for water. Equilibration followed by 150 ns MD simulations of whole systems (receptor + ligand + membrane + ions + water) was performed using NAMD [74] based on the input files generated with the CHARMM-GUI Membrane Builder [75]. Three independent simulations were performed for each system generating a total of 8550 ns trajectory data.

### 4.3. Homology Modeling

The homology model of the fruit fly (*Drosophila melanogaster*) mAChR-A receptor was built using the SWISS-MODEL [76] based on a UniProtKB P16395 (ACM1_DROME) sequence. As a whole sequence model could not be built properly, the residues 300–700 (part of the intracellular loop 3) were removed to obtain the seven transmembrane helices model. The inactive state of the human M1 receptor (PDB code: 5CXV) was used as a template due to the highest similarity and the best scoring.

The quality of the mAChR-A model was validated by PROCHECK [77], ERRAT [78], Verify3D [79] and PROVE [80], all of which belong to the structure analysis-validation online server sponsored by the UCLA-DOE Institute for Genomics and Proteomics. The overall quality factor of ERRAT, expressed as the percentage of the protein for which the calculated error value falls below the 95% rejection limit, equals 98.94. Only three residues exceeded the error value (see SM Figure S9). VERIFY3D validation was passed with 99.66% of the residues having averaged 3D-1D score ≥ 0.2. PROVE validation was passed with no buried outlier protein atoms found. The Ramachandran plot and the all-residue Chi1-Chi2 plots generated by PROCHECK [77] can be found in the SM Figures S10 and S11, respectively.

Molecular docking and dynamics were performed as described above with changed plasma lipid composition. Approximately 200 lipid molecules were used in the following proportions: 38% DOPE, 18% DOPS, 16% DOPC, 13% POPI, 11% SM (CER180), 3% DOPG and 1% PALO 16:1 fatty acid. For the insect model, three repetitions of 500 ns MD simulation each were collected.

### 4.4. Analysis

The analysis and visualization were made using the VMD code [81], Python package [82], and homemade scripts. Snake plots of human M1 and insect mAChR-A were made using the Protter server [29].

A residue–residue contact score (RRCS), an atomic distance-based calculation that quantifies the strength of contact between residue pairs, was calculated with the python script provided by Zhou et al. [43] and further analyzed with the NumPy package.

The percentage of MD frames in which any pairs of M1 residues possibly interacted were calculated using the GetContacts server [52]. MOLEonline [62] and CAVER [63] were used to investigate the water tunnels in protein. For CAVER analysis, trajectories were aligned and processed into a series of PDB snapshots (one frame every 5 ns of MD). The parameters used included the probe radius of 0.9 Å to identify internal tunnels. W^6.48^ was used as a starting point residue for calculations.

## Figures and Tables

**Table 1:** Parametrization of global effects of ligands on averaged contact fractions.

Parameter	DEET	IR3535	Muscarine
GE+	0.276	0.070	0.334
GE−	0.829	1.300	1.682

## Data Availability

Ligand docked structures and the structure of Dm type A mAChR are available from B.N. or W.N. upon request.

## Supplementary Materials

The following are available online at https://www.mdpi.com/article/10.3390/molecules27103280/s1, Figure S1. Molecular docking of DEET and IR3535 to the human M1 GPCR (PDB code: 5CV) together with the SMINA scoring function (SSF) affinity decomposition. Figures S2–S5. Molecular docking of ligands (DEET, IR3535, muscarine, atropine, BQCA, Oxotremorine-M, pirenzepine, BQZ-12) to the human M1 GPCR (PDB code: 5CV). SMINA scoring function (SSF) shows the minimized affinity of the ligand to the receptor. Figure S6. Histogram plots for residue–residue contact scores (RRCSs) of human M1 receptor in APO form and bound with DEET, IR3535, or muscarine. Figure S7a,b. Histogram plots for residue–residue contact scores (RRCSs) of human M1 receptor in APO form and with IR3535, IR3535 together with BQCA and with bitopic BQCA-azo-IR3535 ligand bound. Figure S8. BQCA-azo-IR3535 interaction with human M1 receptor and insect mAChR-A model. Figures S9–S11. Homology model assessment. Table S1. SSF values of repellents docking to the active structures.

## Sample Availability

Samples of the compounds are not available from the authors.
