# Supplementary material for: In Search of Synergistic Insect Repellents: Modeling of Muscarinic GPCR Interactions with Classical and Bitopic Photoactive Ligands
Source: Molecules. 2022 May 20;27(10):3280. doi: 10.3390/molecules27103280 (PMC9147842; doi:10.3390/molecules27103280)
Supplement: Supplementary file 1 [file molecules-27-03280-s001.zip › molecules-1709172-supplementary.pdf]

Supplementary Materials

Article

In search for synergistic insect repellents: Modeling of muscarinic GPCRs interactions with classical and bitopic photoactive ligands.

Beata Niklas[1], Bruno Lapied[2], Wieslaw Nowak[1,*]

[1] Institute of Physics, Faculty of Physics, Astronomy and Informatics, Nicolaus Copernicus University in To-run, Torun, Poland

[2] Laboratoire Signalisation Fonctionnelle des Canaux Ioniques et des Récepteurs (SiFCIR), UPRES EA 2647, USC INRA 1330, SFR QUASAV 4207, UFR Sciences, Université d'Angers, 2 boulevard Lavoisier, 49045, Angers Cedex, France.

* Correspondence: wiesiek@umk.pl

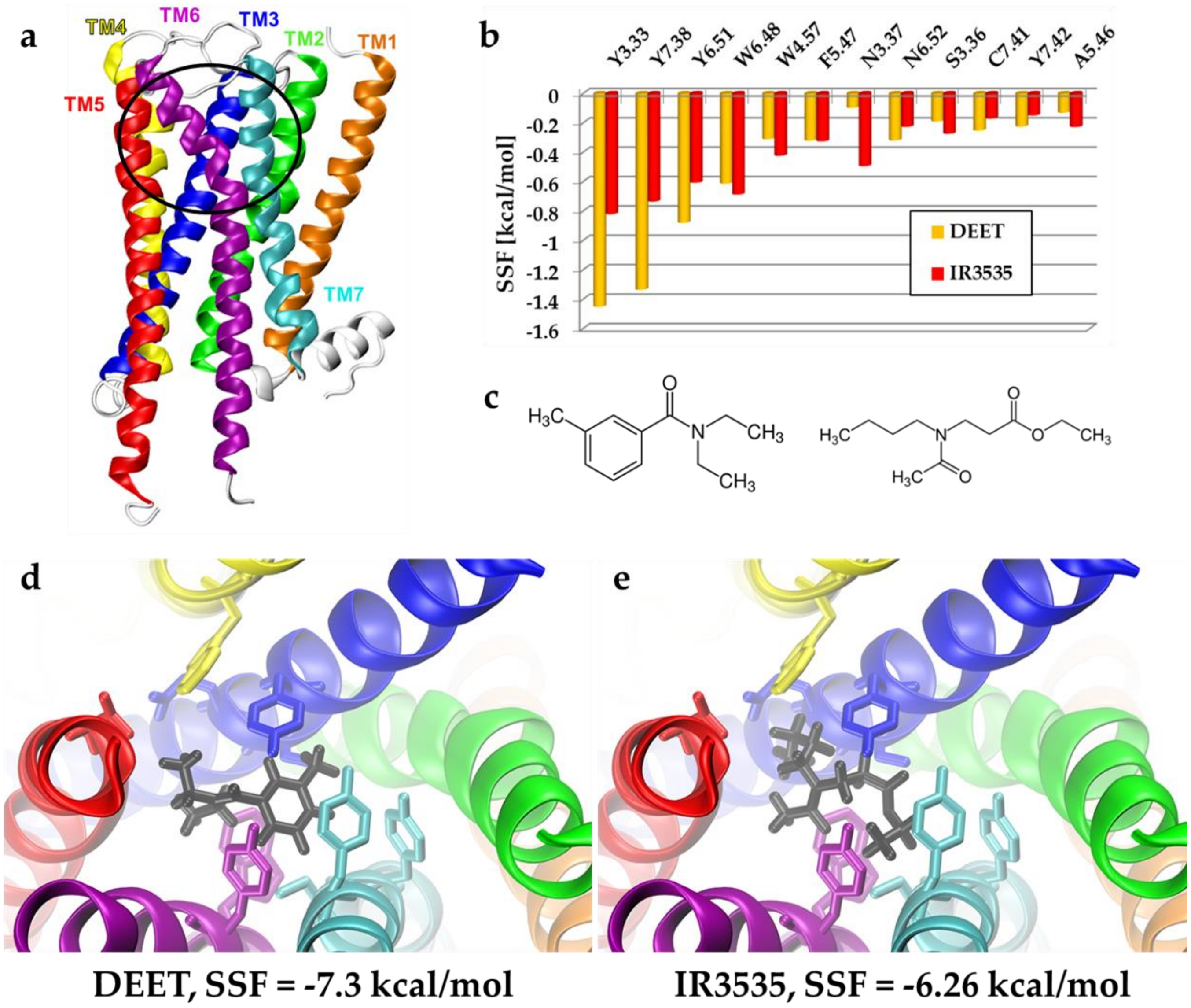

Figure S1. SMINA molecular docking of insect repellents to the human M1 muscarinic receptor. a) Model of human M1 receptor based on the X-ray structure (PDB code: 5CXV) with orthosteric binding site region marked with a black oval. b) Docking energy decomposition presented as SMINA scoring function (SSF) shows interacting residues of the M1 orthosteric binding site c) Structures of DEET (left) and IR3535 (right). The best scored docking poses of DEET (d) and IR3535 (e) are shown in a top views.

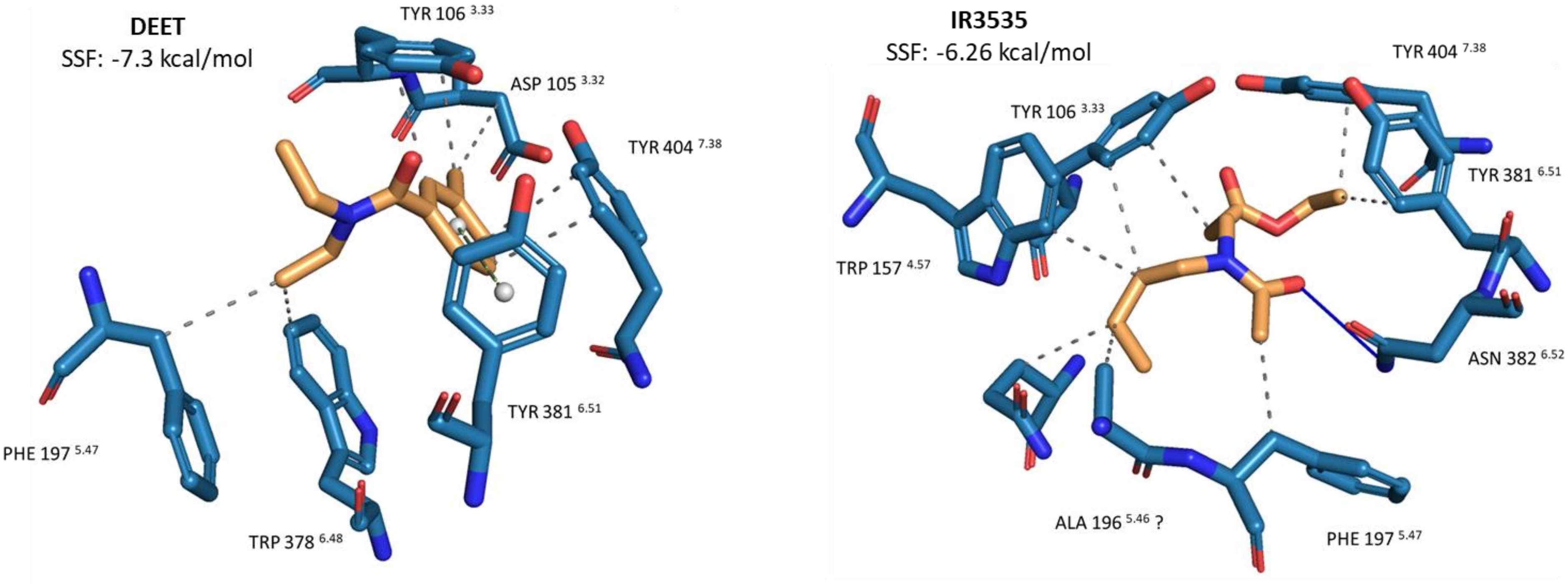

Figure S2. Molecular docking of DEET (left) and IR3535 (right) to the orthosteric site of human M1 GPCR (PDB code: 5CV). SMINA scoring function (SSF) shows the minimized affinity of ligand to the receptor.

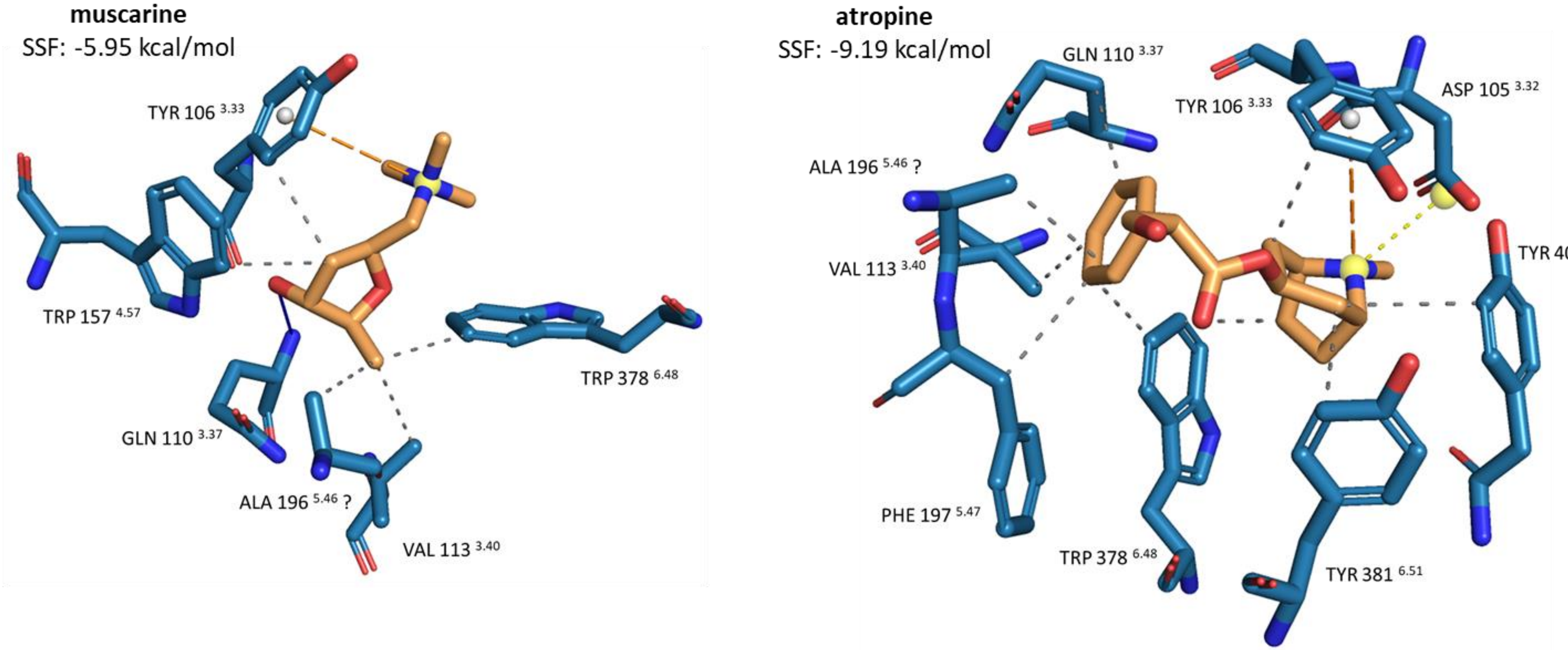

Figure S3. Molecular docking of agnonist muscarine (left) and antagonist atropine (right) to the orthosteric site of human M1 GPCR (PDB code: 5CV). SMINA scoring function (SSF) shows the minimized affinity of ligand to the receptor.

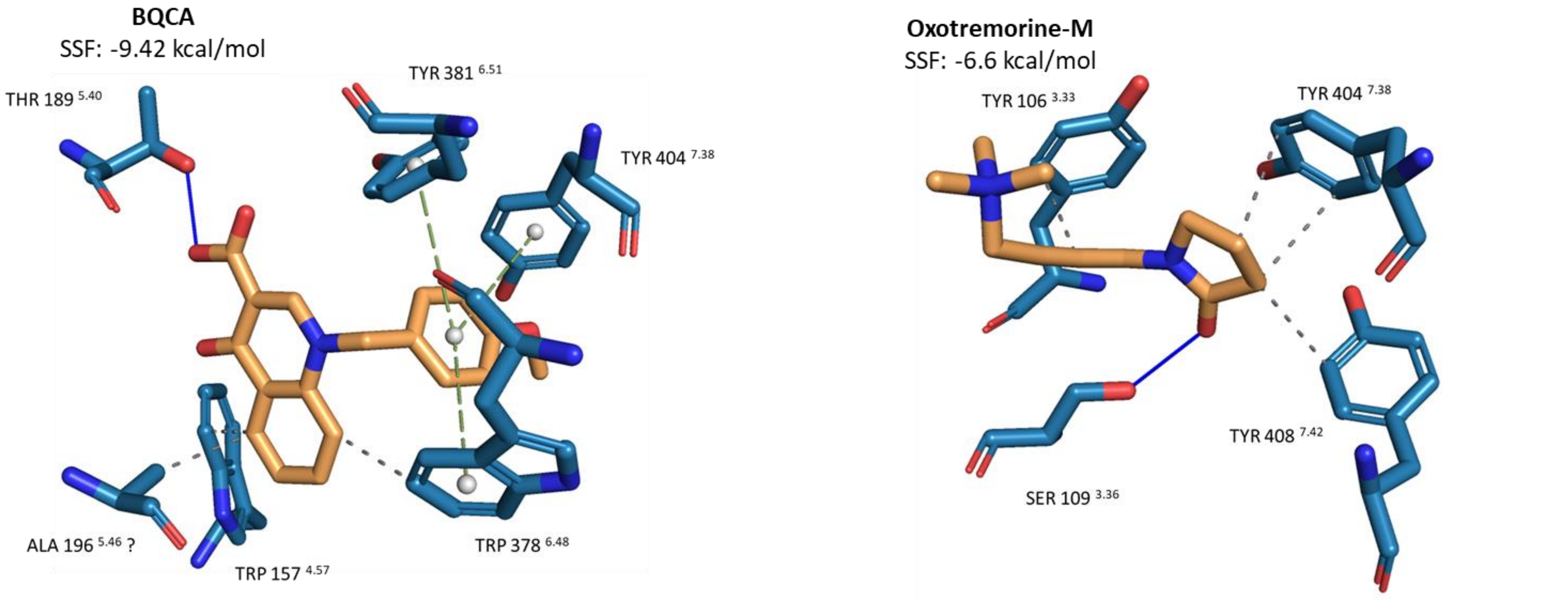

Figure S4. Molecular docking of BQCA (left) and Oxotremorine-M (right) to the orthosteric site of human M1 GPCR (PDB code: 5CV). SMINA scoring function (SSF) shows the minimized affinity of ligand to the receptor.

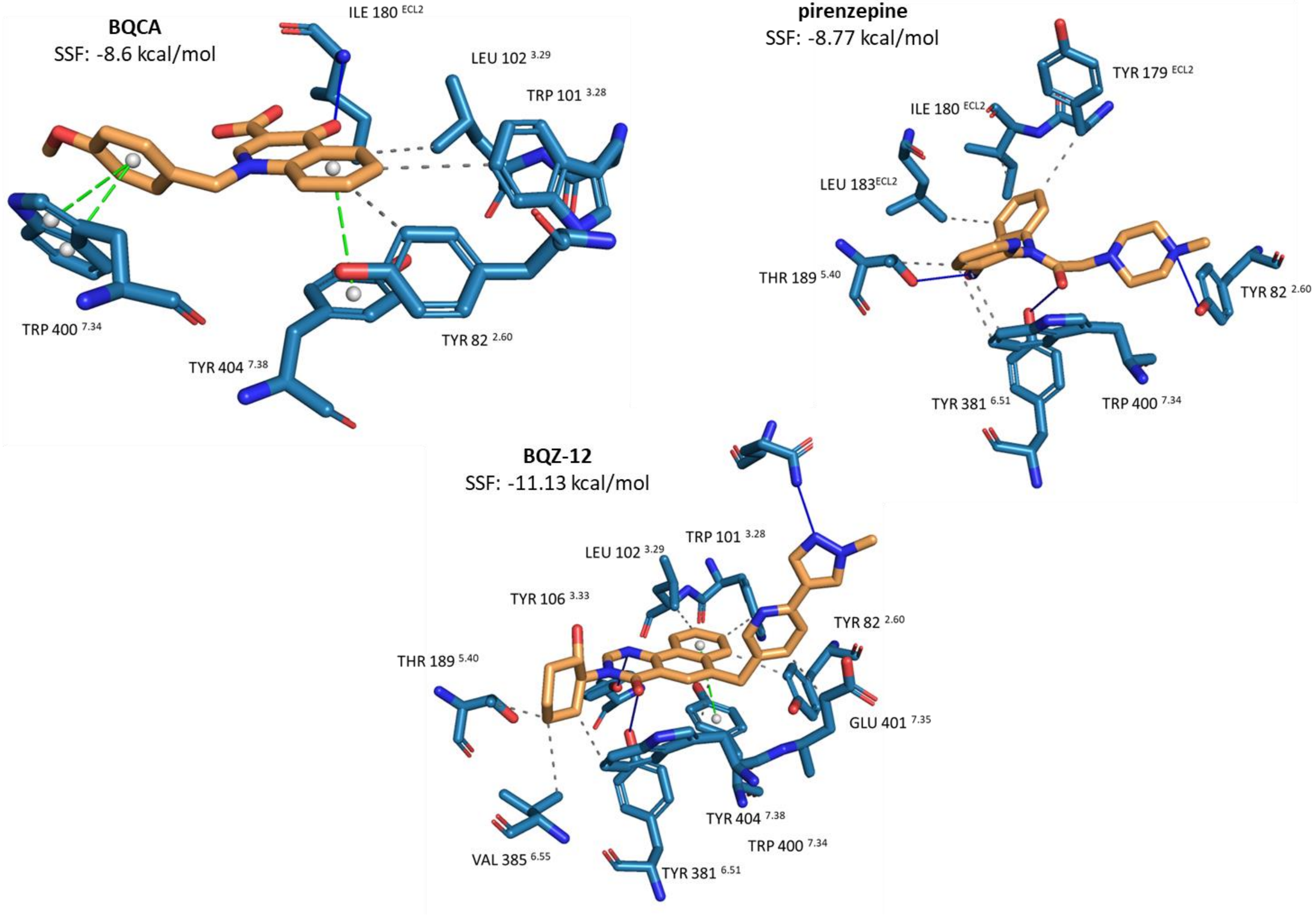

Figure S5. Molecular docking of BQCA (upper left), pirenzepine (upper right) and BQZ-12 (lower panel) to the allosteric site of human M1 GPCR (PDB code: 5CV). SMINA scoring function (SSF) shows the minimized affinity of ligand to the receptor.

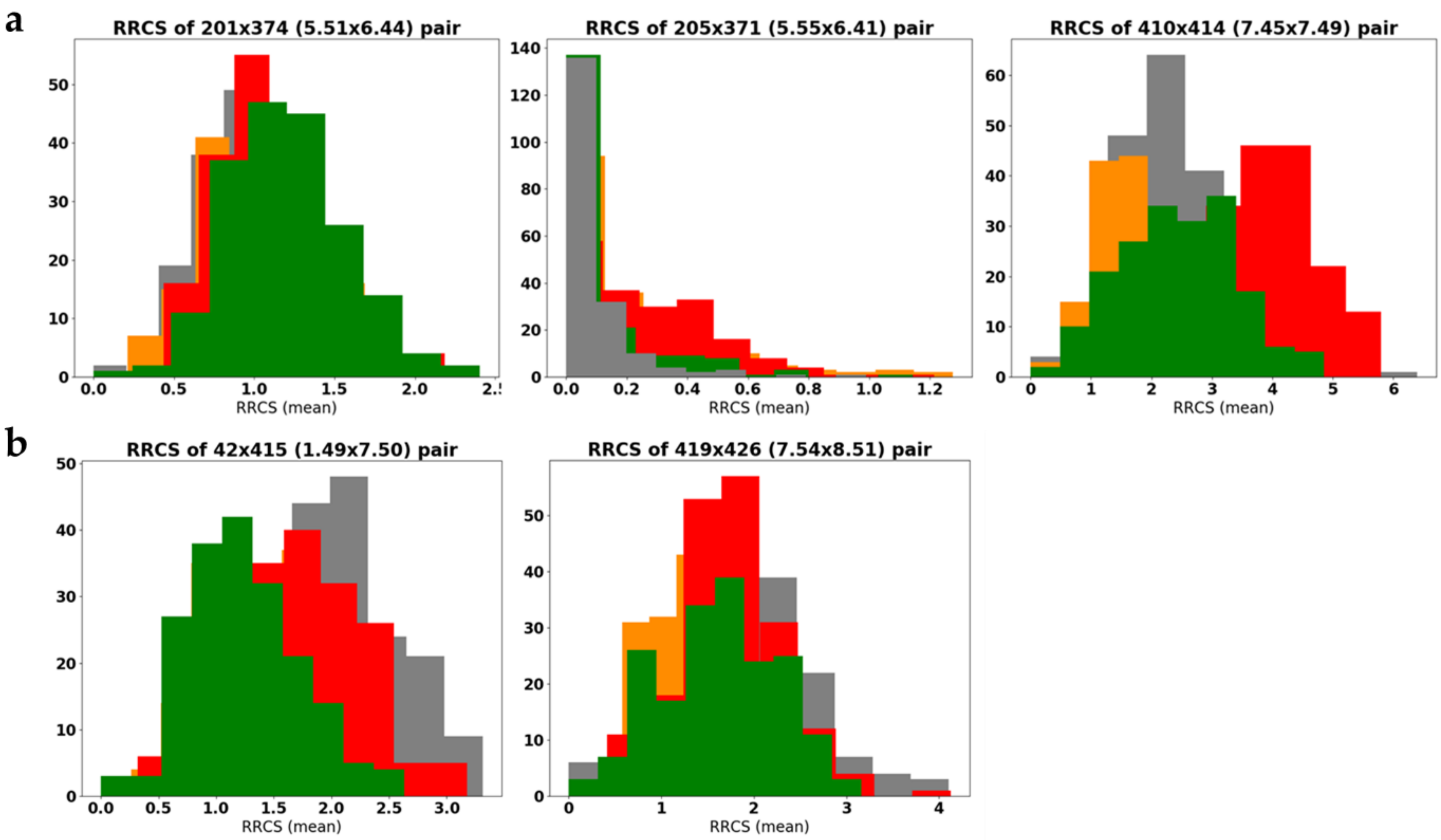

Figure S6. Histograms of residue-reside contact scores (RRCSs). Sampling was 1 frame/0.8 ns of 150 ns MD simulation (average of 3 repetitions). Contacts that increase (a) and decrease (b) RRCS are shown for M1 apo receptor in grey, M1 with muscarine in green, M1 with DEET in orange and M1 with IR3535 in red.

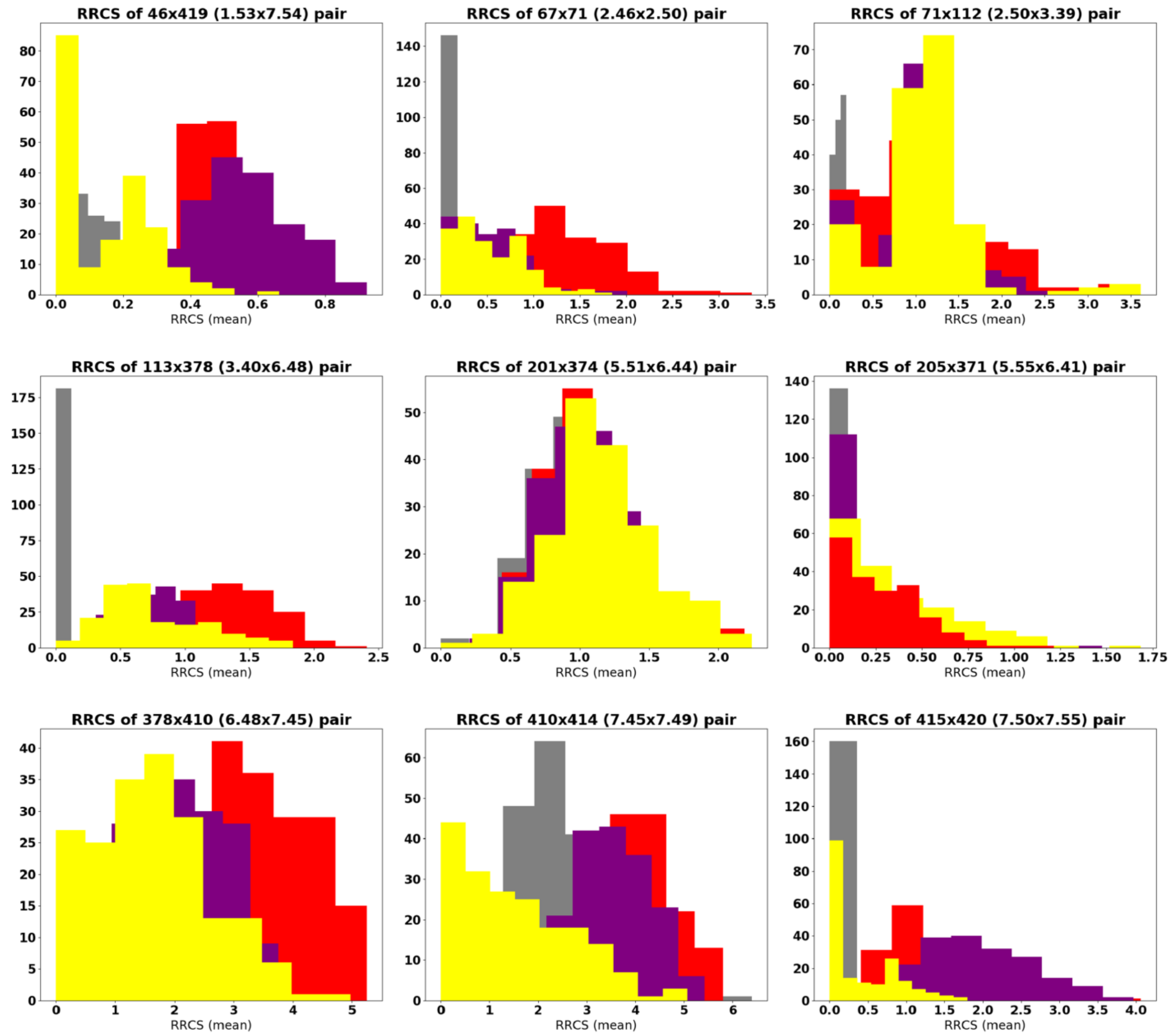

Figure S7a. Histograms of residue-reside contact scores (RRCSs) calculated for 1 frame/0.8 ns of 150 ns MD simulation (average of 3 repetitions). Contacts that increase RRCS upon activation are shown with M1 apo receptor in grey, M1 with IR3535 in red, M1 with IR3535 in the orthosteric site and BQCA in the allosteric site in purple and M1 with BQCA-azo-IR3535 in yellow.

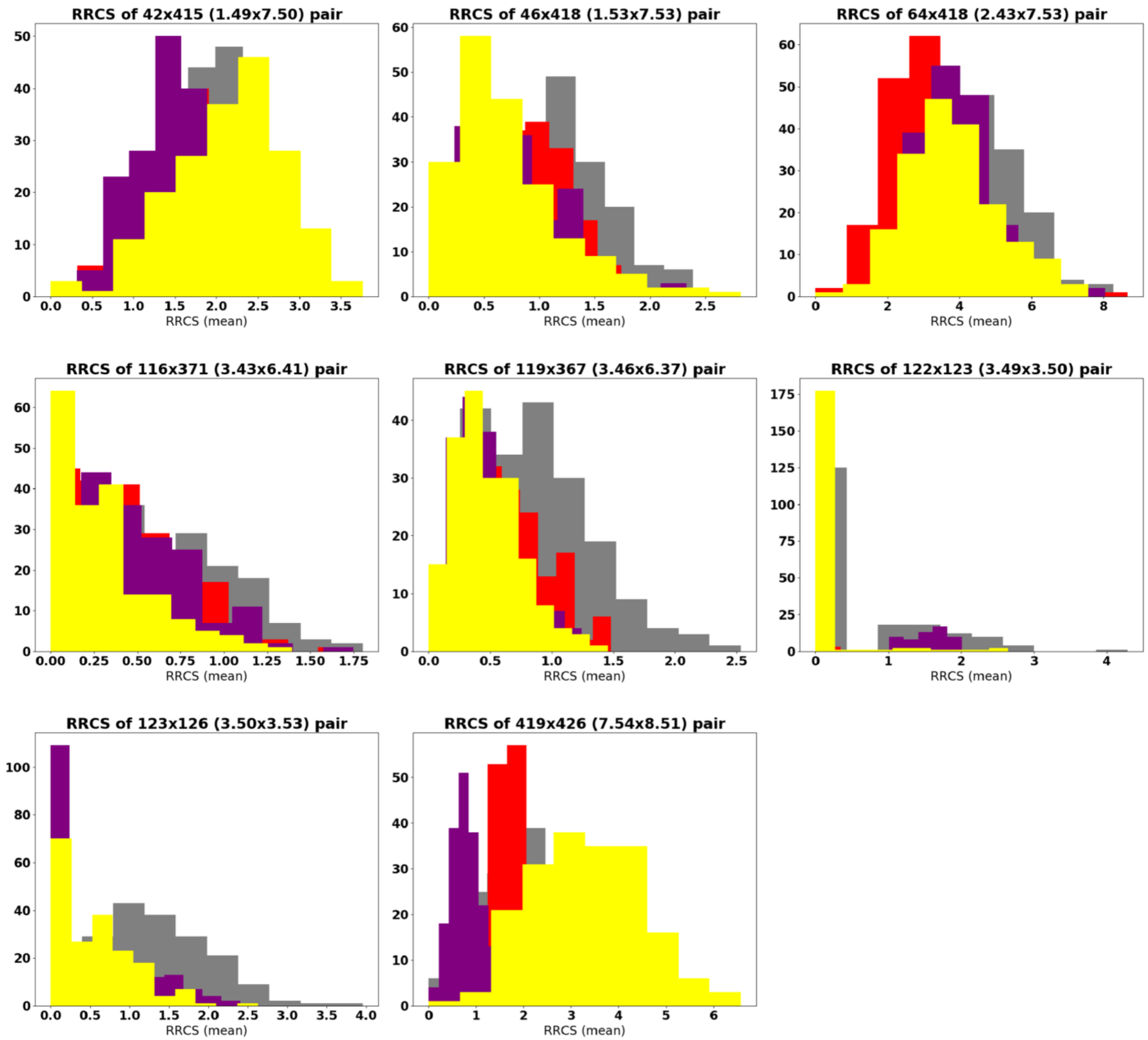

Figure S7b. Histograms of residue-reside contact scores (RRCSs) calculated for 1 frame/0.8 ns of 150 ns MD simulation (average of 3 repetitions). Contacts that decrease RRCS upon activation are shown with M1 apo receptor in grey, M1 with IR3535 in red, M1 with IR3535 in the orthosteric site and BQCA in the allosteric site in purple and M1 with BQCA-azo-IR3535 in yellow.

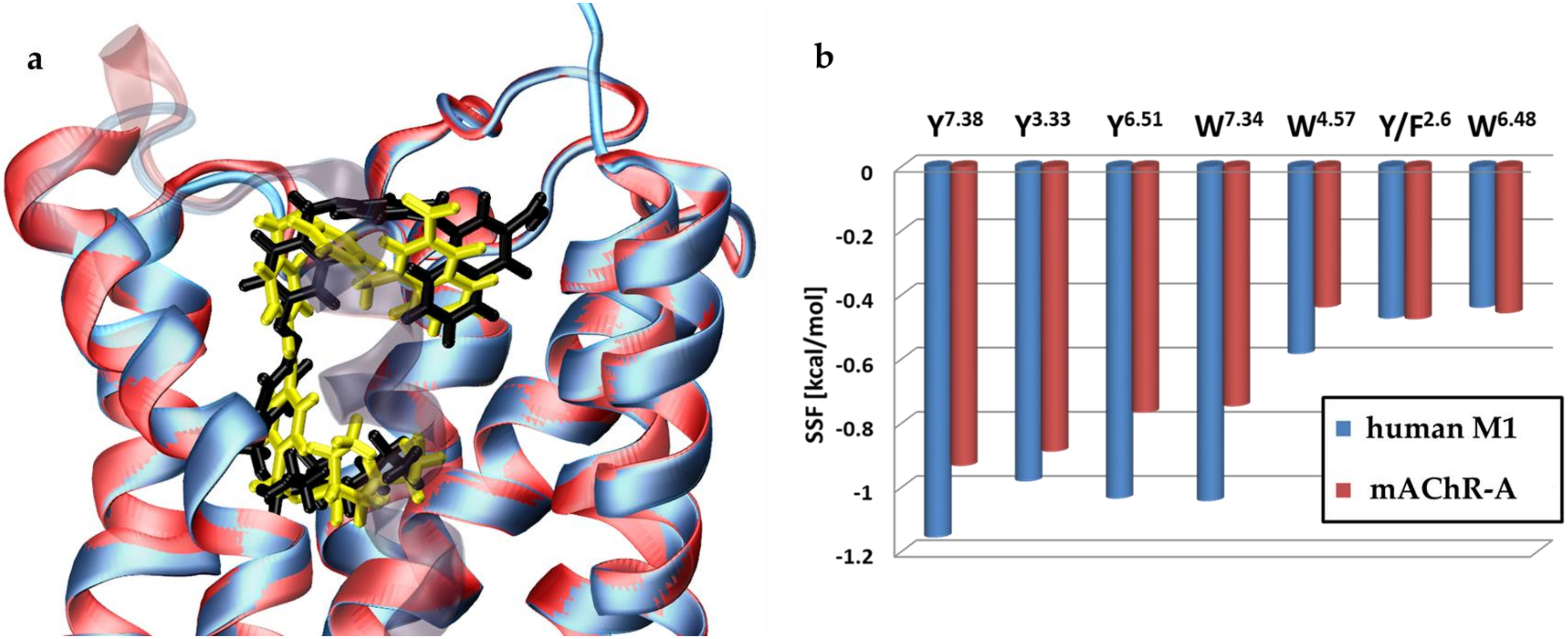

Figure S8. BQCA-azo-IR3535 interaction with human M1 receptor (dark blue) and insect mAChR-A model (red). a) The lowest energy pose of BQCA-azo-IR3535 in human M1 (ligand in yellow) and in insect mAChR-A (ligand in black). b) Docking energy decomposition presented as a SMINA scoring function (SSF) shows the most important residues in binding to both receptors).

Table S1. The minimized affinity (Smina Scoring Function in kcal/mol) of the ligand to the human M1 receptor and the insect model build using given template.

		5CXV	6OIJ	6ZG9
Human M1	DEET	-7.3	-6.30	-7.55
	IR3535	-6.26	-6.43	-6.03
	BQCA-azo-IR3535	-11.35	-8.24	-10.29
Insect mAChR-A (model)	DEET	-7.19	-6.07	
	IR3535	-6.08	-6.31	
	BQCA-azo-IR3535	-11.97	-8.56	

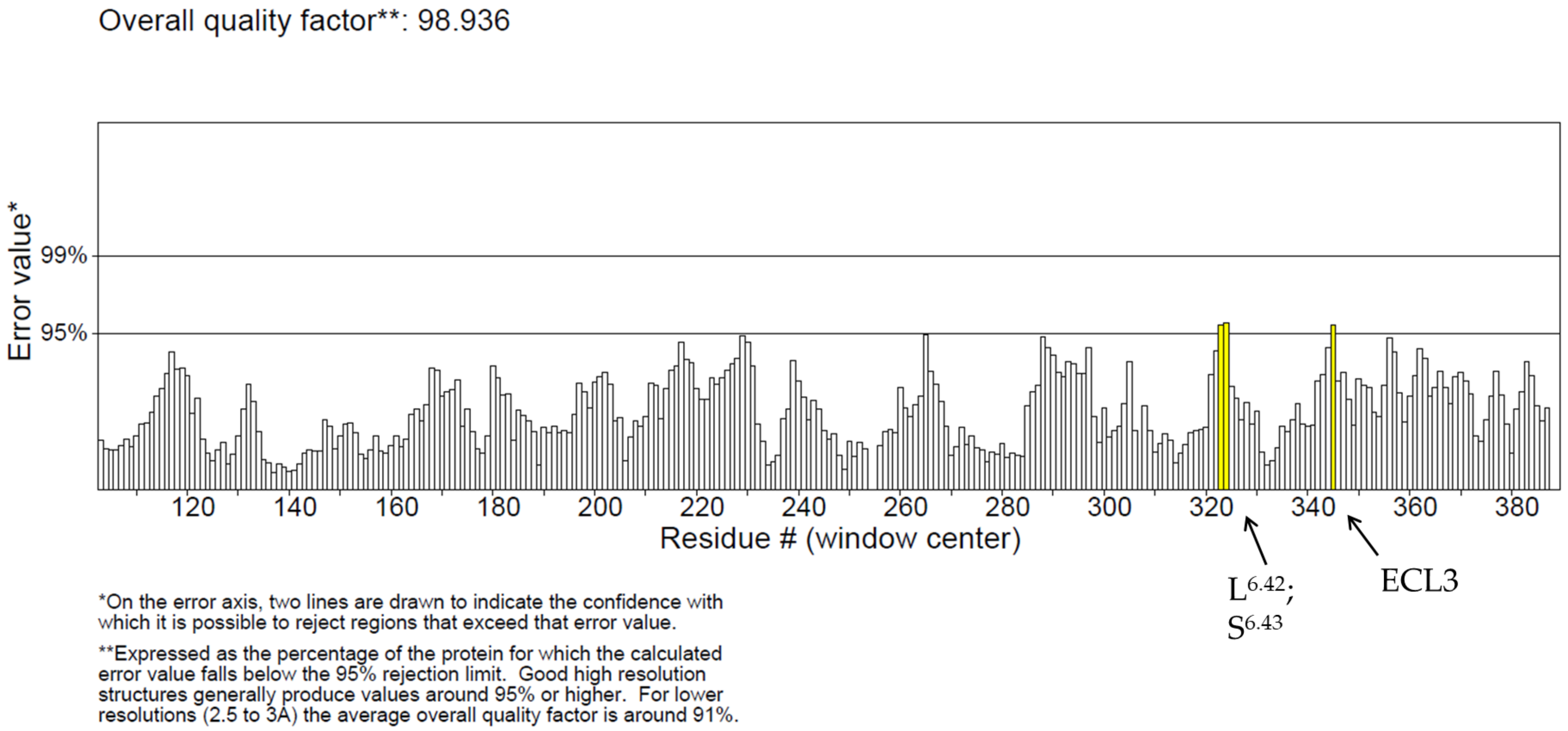

Figure S9. Homology model assessment of *Drosophila melanogaster* mAChR-A made using ERRAT server [1]. The overall quality factor, expressed as the percentage of the protein for which the calculated error value falls below the 95% rejection limit, equals 98.94.

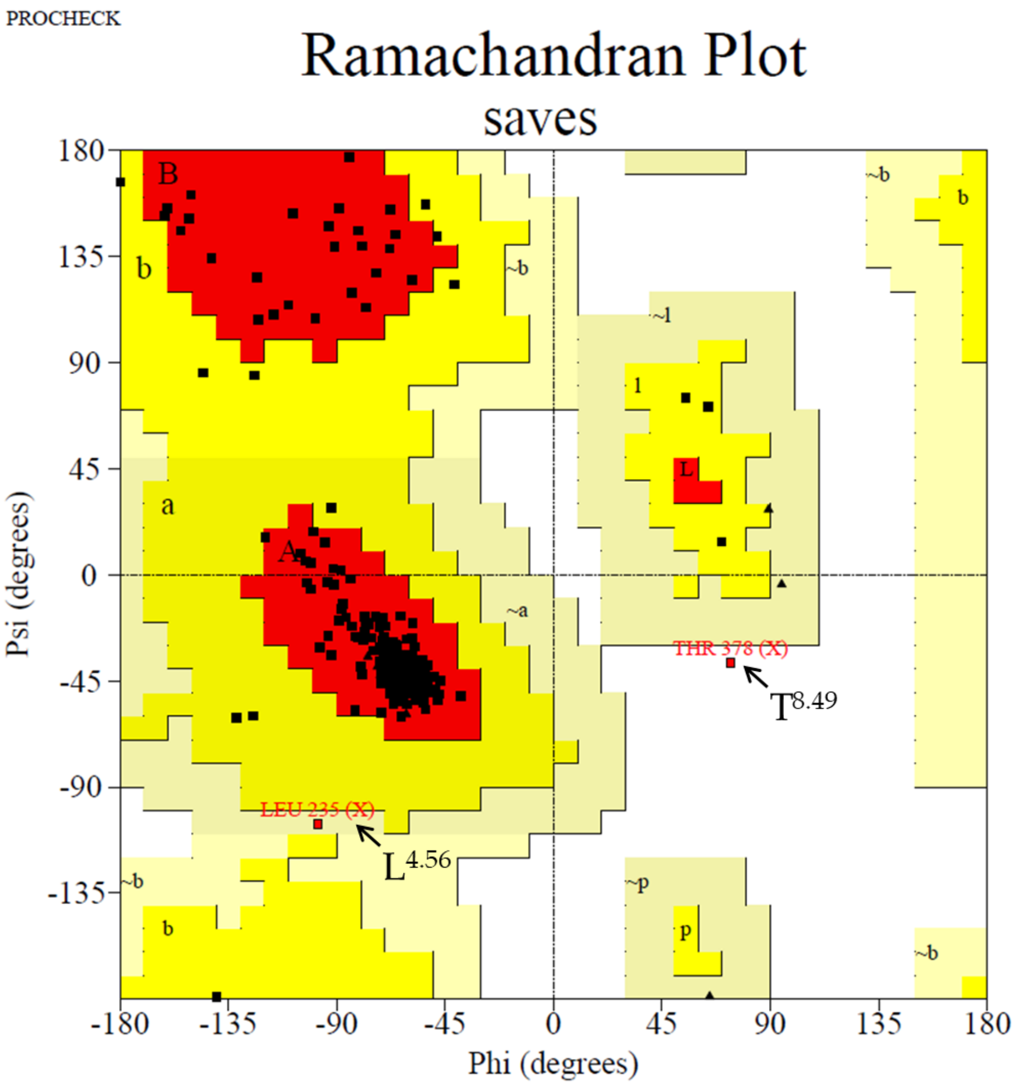

Plot statistics

Residues in most favoured regions [A,B,L]	257	94.8%
Residues in additional allowed regions [a,b,l,p]	12	4.4%
Residues in generously allowed regions [~a,~b,~l,~p]	1	0.4%
Residues in disallowed regions	1	0.4%
	----	------
Number of non-glycine and non-proline residues	271	100.0%
Number of end-residues (excl. Gly and Pro)	123	
Number of glycine residues (shown as triangles)	9	
Number of proline residues	13	

Total number of residues	416	

Based on an analysis of 118 structures of resolution of at least 2.0 Angstroms and R-factor no greater than 20%, a good quality model would be expected to have over 90% in the most favoured regions.

Figure S10. Ramachandran Plot of *Drosophila melanogaster* mAChR-A homology model made using PROCHECK server [2].

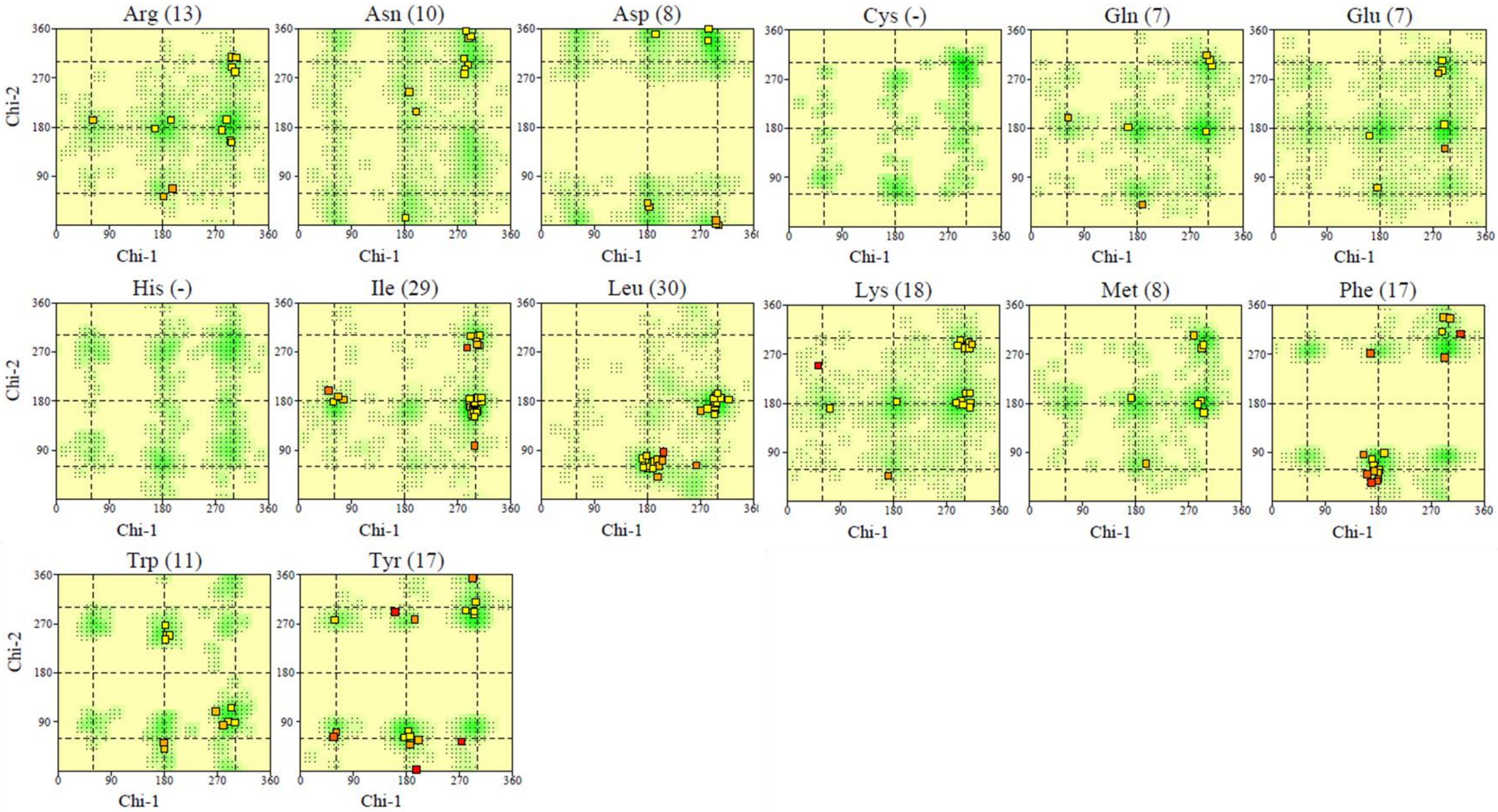

Figure S11. All-residue Chi1-Chi2 plots of *Drosophila melanogaster* mAChR-A homology model made using PROCHECK server [2].

1. Colovos, C. and T.O. Yeates, *Verification of protein structures: patterns of nonbonded atomic interactions.* Protein science, 1993. **2**(9): p. 1511-1519.
2. Laskowski, R.A., et al., *PROCHECK: a program to check the stereochemical quality of protein structures.* Journal of applied crystallography, 1993. **26**(2): p. 283-291.
